# Influence of professional background on assessment of simulated cardiopulmonary resuscitation videos in an observational study

**DOI:** 10.1038/s41598-025-12306-x

**Published:** 2025-07-29

**Authors:** Thomas Wetzel, Jan Wienstroer, Saša Sopka, Hanna Schroeder, Marc Felzen, Jörg C. Brokmann, Christopher Plata

**Affiliations:** 1 Department for Acute and Emergency Medicine, Medical Faculty RWTH, Aachen University, Pauwelsstrasse 30, 52074 Aachen, Germany; 2 Institute for Medical Informatics, Medical Faculty, RWTH Aachen University, Pauwelsstrasse 30, 52074 Aachen, Germany; 3 Department of Anaesthesiology, Medical Faculty, RWTH Aachen University, Pauwelsstrasse 30, 52074 Aachen, Germany; 4 AIXTRA-Competence Center for Training and Patient Safety, Medical Faculty, RWTH Aachen University, Pauwelsstrasse 30, 52074 Aachen, Germany; 5 Aachen Institute for Rescue Management & Public Safety, City of Aachen and Medical Faculty, RWTH Aachen University, Pauwelsstrasse 30, 52074 Aachen, Germany; 6 Center for Emergency medicine, University Hospital RWTH Aachen, Pauwelsstrasse 30, 52074 Aachen, Germany

**Keywords:** V-CPR, Video-CPR, Video-assisted CPR, VA-CPR, Emergency medical dispatcher, Dispatcher profession, Dispatcher requirements, Layperson CPR, Bystander CPR, Cardiovascular diseases, Medical research

## Abstract

Video-assisted dispatcher support in cardiopulmonary resuscitation (VA-CPR) has shown beneficial effects on CPR quality. This study examines the influence of professional background on the ability to identify typical errors in CPR performance using video-based assessments. Within this simulation-based observational study, 61 participants (31 EMS personnel, 30 emergency physicians) evaluated nine video sequences showing simulated CPR or ventilation. Participants were grouped by profession, not randomly. The primary endpoint was the correct identification of expert-defined errors in the presented videos, analyzed in relation to professional background. Evaluation accuracy for CPR and ventilation videos was examined in relation to participant characteristics. Overall, *n* = 427 CPR videos were correctly classified in 73.3% of cases by EMS personnel and 75.7% by emergency physicians (β = 0.370, SE = 0.297, 95% CI: −0.21 to 0.95, *p* = 0.213). Ventilation scenarios (*n* = 122) were correctly classified in 93.5% (EMS) and 98.3%, (EP) (β = 4.50, SE = 6.73, 95% CI: −8.82 to 17.82, *p* = 0.505). The models assessing classification accuracy for CPR and ventilation did not reach statistical significance (p_CPR_=0.869 and p_ventilation_=0.183), and none of the tested predictors were significantly associated with evaluation accuracy. No significant differences in evaluation accuracy for CPR and ventilation videos were observed between professional groups or across tested participant characteristics.

**Supplementary Information:**

The online version contains supplementary material available at 10.1038/s41598-025-12306-x.

## Introduction

Out-of-hospital cardiac arrest (OHCA) represents a major public health concern and was reported as the third leading cause of death in Europe in 2021. Its incidence is estimated to range from 67 to 170 cases per 100,000 inhabitants^1^. The average survival rate at hospital discharge remains low, at approximately 10%. One of the key factors associated with improved outcomes is the prompt initiation of high-quality cardiopulmonary resuscitation (CPR) by laypersons^2,3^. Currently, 80% of European countries offer dispatcher-assisted telephone CPR (T-CPR)^1^. Despite the inherent limitations of audio-only communication, telephone-based CPR remains the standard method for guiding and supporting lay rescuers during resuscitation efforts.

The COVID-19 pandemic has significantly accelerated the adoption of video telephony. Within the first year of the pandemic, the proportion of physician-patient contacts conducted via video consultation doubled from 11 to 22%^4^. Moreover, telemetry and video communication are already established components of prehospital emergency care^5–7^. These technologies facilitate real-time support for emergency medical service (EMS) personnel and enable the direct transmission of medical data^7^. However, video-assisted CPR has not yet been widely implemented and is currently utilized by only a limited number of dispatch centers. Nevertheless, evidence suggests that this approach may offer substantial benefits^8–11^. The inclusion of visual information enables dispatchers to deliver more precise instructions and provide corrective feedback regarding compression rate and depth, correct hand placement, and full chest recoil after each compression. Furthermore, four out of five dispatchers support the use of this technology and report perceived benefits in terms of guidance and overall quality improvement^12^.

In Germany, dispatch centers are primarily staffed by certified paramedics. In addition, physician-supported telemedicine systems are increasingly employed to deliver video-based medical guidance, positioning both paramedics and emergency physicians as key groups for delivering instructions in video-assisted CPR. Despite this, data on the prerequisites for implementing video-assisted CPR (VA-CPR) within the existing prehospital emergency infrastructure remain scarce. It also remains unclear whether professional background influences the evaluation of CPR performance in VA-CPR. We thus tested the primary hypothesis that the correct evaluation of chest compression and ventilation video sequences depends on the evaluator’s profession. Secondarily, we tested whether classification of specific CPR and ventilation scenarios differs between professions.

## Methods

### Study design

**Fig. 1:**
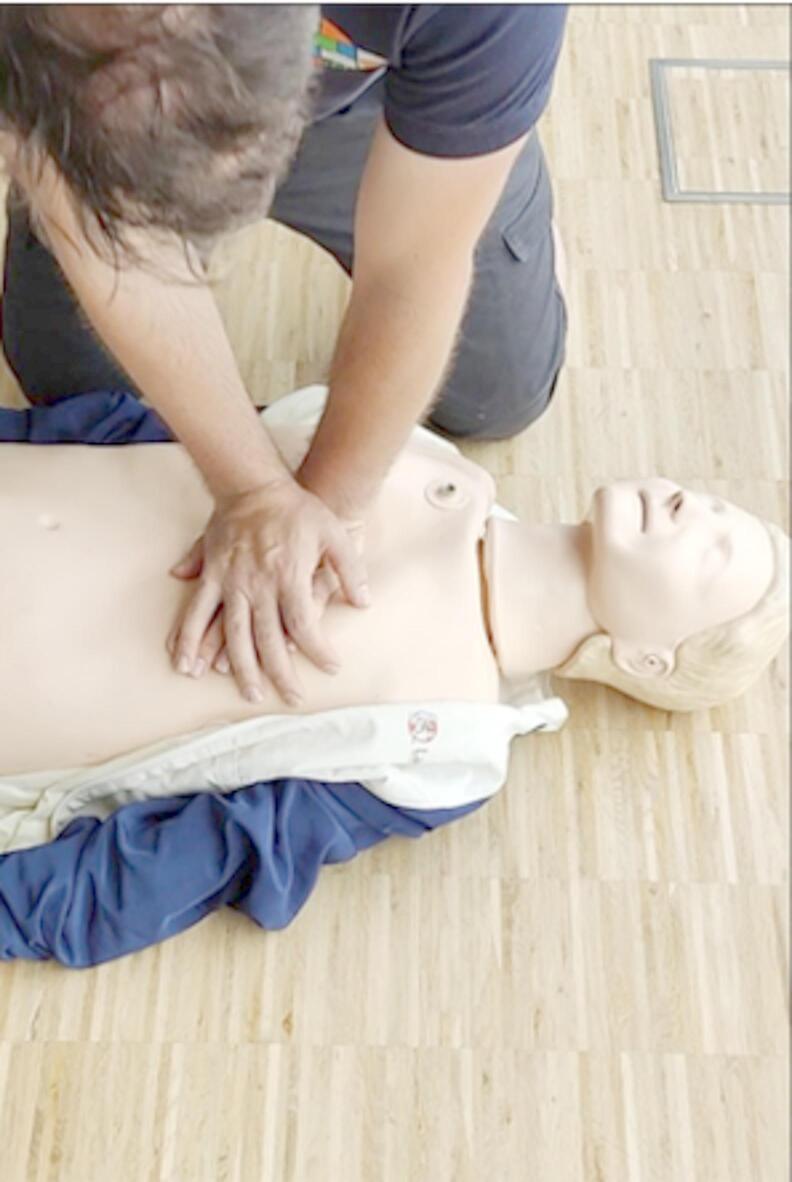
Screenshot of a video sequence with lateral camera perspective, as presented during the evaluation phase to both professional groups.

The study was approved by the local Ethics Committee of the Medical Faculty of the RWTH University Aachen, Germany (approval number 22–164, 5th of July 2022) and was registered at the German Clinical Trial Register (Registration number DRKS00029696) prior to inclusion of the first subject. All research was performed in accordance with relevant guidelines and regulations, and in accordance with the Declaration of Helsinki. Informed consent was obtained from all participants. Informed consent was obtained to publish the image (Fig. 1) in an online open access publication. During this simulation-based observational study, 61 participants evaluated seven video sequences of CPR and two video sequences of ventilation on a screen. Correct classification of the video sequences was evaluated depending on the participants’ profession, hence blinding was not applicable. This study was supported by the Center for Acute and Emergency Medicine and the Department of Emergency Medicine at RWTH Aachen University. Reporting was carried out in accordance with the TREND reporting guidelines^13^.

### Video sequences and recruitment

**Fig. 2:**
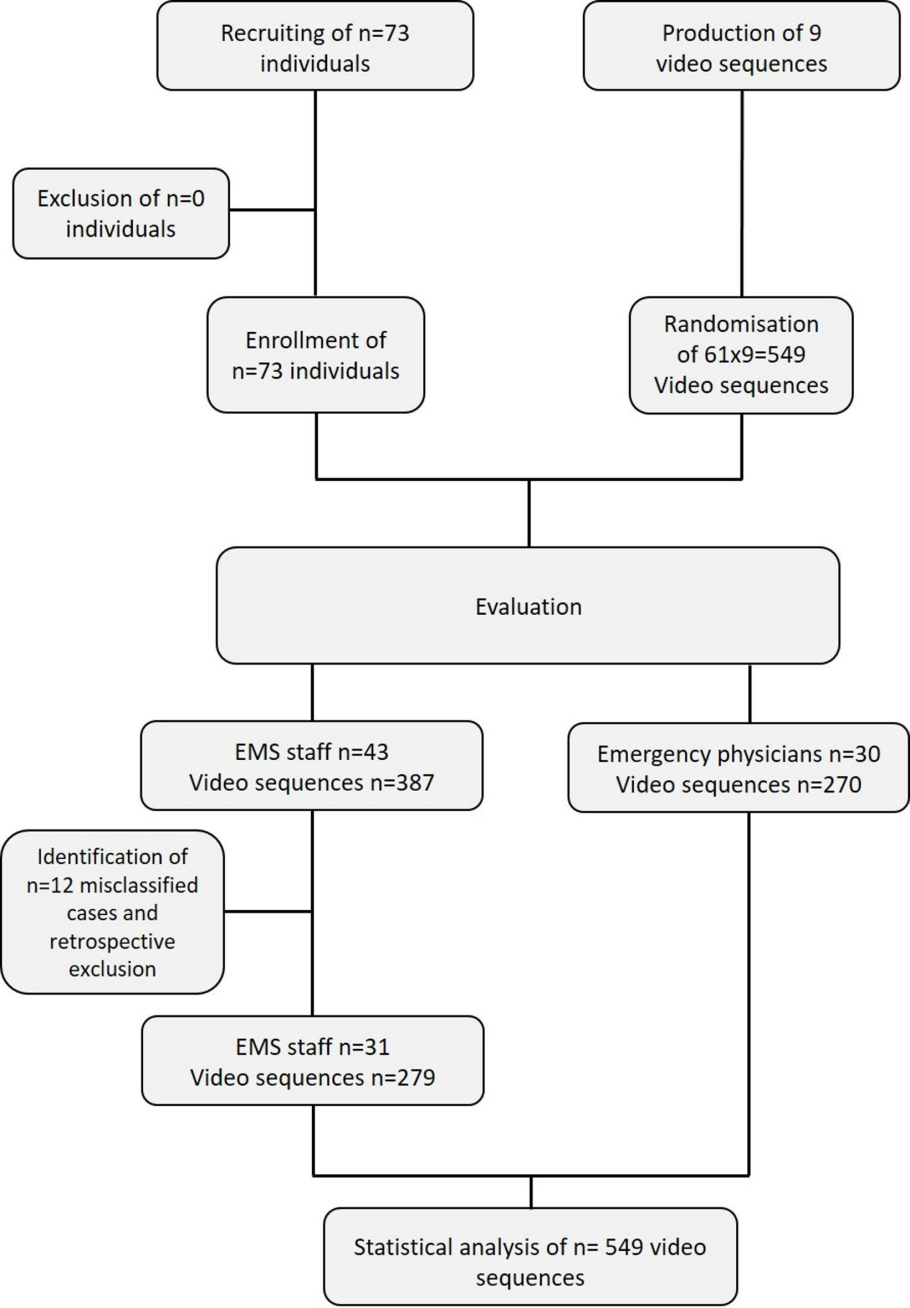
Study flow chart. A total of 12 out of 73 participants were retrospectively excluded after enrollment, as they did not fulfill the predefined inclusion criteria. EMS: emergency medical service.

The initial step involved the recording of nine video sequences depicting a simulated CPR on a manikin (Resusci Anne CPR, Laerdal Medical GmbH, Puchheim, Germany). Seven sequences showed different performances of chest compressions: correct CPR, high compression rate (140 min-1), incomplete thorax release (remaining depth > 20 mm), increased compression depth (> 60 mm), low compression rate (80 min-1), superficial compression depth (< 40 mm) and wrong hand position (epigastric compression point). Another two videos showed an insufficient and a sufficient mouth-to-nose ventilation. Each video displayed only one of the predefined scenarios. The duration of each clip was 10 s. Each video was recorded using a smartphone (OnePlus 8 T, OnePlus, Shenzhen, China) with a resolution of 3.840 × 2.160 pixels and a rate of 60 Hz. The camera was mounted on a tripod in a tilt angle of 43 degrees with a distance of 165 cm from lens to breastbone of the manikin (Fig. 1). The camera was positioned on the opposite side of the layperson performing CPR for best conditions possible^14^ (Fig. 2). For the CPR scenarios, quality validation was assured by using the internal depth control display of the manikin and a metronome (‘The Metronome’ App, Soundbrenner limited, Berlin, Germany), being invisible in the final video sequence. Prior to the commencement of the study, the depicted error was evaluated by two independent experts. In the event that both experts concurred that the video was suitable for accurately displaying the specific CPR and ventilation performance, the video was included. Subsequently, medical professionals were recruited through convenience sampling from eligible EMS personnel and emergency physicians associated with the University Hospital Aachen, Germany and the Aachen Fire Brigade Rescue Station, including the affiliated Aachen School for Firefighters. Every participant was informed about the purpose and gave written informed consent prior to inclusion. Data privacy for all participants was obtained and pseudonymized data was used for further analyses. Personal data including age, gender, profession, professional experience and number of passed CPR courses was obtained using a standardized questionnaire (see supplements). The study was conducted at the Emergency Department and the Department of Anesthesiology at the University Hospital of the RWTH University of Aachen, Germany and at the Institute of rescue medicine and civil security (ARS) of Aachen (Aachen, Germany).

### Inclusion and exclusion criteria

Inclusion criteria were voluntary participation, age between 18 and 65 years and active employment as paramedic or emergency physician (EP). The participants were not necessarily active dispatchers, but were selected based on their clinical background as either EMS personnel or emergency physicians. The age range of 18 to 65 years was chosen to represent the active professional population. Exclusion criterion was refusal.

### Evaluation process

Recruitment and data collection took place from October 2022 until April 2023. Following the non-blinded study design, participants were allocated to one of two study groups according to their profession of either EMS-staff or emergency physicians. There was no randomization of the participants. During the evaluation process, each participant evaluated video sequences using a standardized questionnaire with multiple-choice answers regarding the categories compression rate, compression depth, compression point and release after compression. A video was considered correctly evaluated if the error depicted in the video matched the participant’s classification. Participants were informed that each video displayed one predominant error. However, they were not restricted from selecting multiple answers if they perceived more than one issue. Prior to the assessment, all participants received a brief standardized introduction to the evaluation rubric but did not undergo any specific training or coaching on how to interpret it. The order of video presentation was randomized individually for each participant to minimize order effects using a computer-generated block randomization list (MS Excel, Microsoft Corporation, Redmond, Washington, USA). Video sequences were displayed in MP4 format via Windows Media Player on a Lenovo ThinkPad laptop. The display was a 14-inch screen with a resolution of 1920 × 1080 pixels, manufactured by Sony Group Corporation (Minato, Tokyo, Japan), Microsoft Corporation (Redmond, Washington, USA), and Lenovo Group Limited (Quarry Bay, Hong Kong). To avoid distracting background noises, video sequences were muted for the evaluation. An additional questionnaire (see supplements, form S1) captured specific personal characteristics of the participants (sex, age, profession, specialization, professional experience, number of passed CPR courses). To avoid inter-participant bias, each evaluation was conducted individually, and participants were explicitly instructed not to share study content or discuss the videos with others during the data collection period.

### Outcomes

The primary outcome parameter was the correct evaluation of the presented CPR or ventilation video sequence depending on the profession of the evaluator. The secondary outcome parameters include the correct evaluation of each single error and the false-positive indication of non-existent errors. Finally, we examined the impact of the professional experience, special training, age and sex of every participant on the evaluation of the videos.

### Statistical analysis

Due to the lack of prior information regarding the expected distribution of differences and the standard deviations of the outcome measures, no formal sample size calculation was performed. However, based on insights from previous studies^14,15^ and considering the exploratory nature of the investigation, a target sample size of 30 participants per study group was deemed appropriate. Also, this number seemed manageable and sufficient for the following logistic regression analysis. Descriptive analyses present numbers and percentages for categorical variables. Thereby, normally distributed data is presented as mean ± standard deviation (SD). Quantitative data not following normal distribution is presented as median and interquartile range (IQR). To compare the backgrounds of participants in the two groups, the t-test for independent samples was applied for continuous variables with approximately normal distribution (age), while the Mann–Whitney U test was used for continuous variables that did not meet the assumption of normality (professional experience, number of passed CPR courses). Categorical variables were compared using the chi-square test (gender). Normality was assessed visually and by using the Shapiro–Wilk test. Statistical analysis of the primary endpoint was performed using a mixed-effects logistic regression model. Secondary endpoints were analyzed descriptively. To further explore potential influencing factors, we constructed a linear mixed-effects model with the total number of correct responses per participant in the CPR video scenarios as the dependent variable, examining the effects of age, sex, professional experience, and number of passed CPR courses on participants’ evaluation performance. This approach accounts for both fixed effects of predictors and random effects to handle potential clustering or repeated measures within the data. We considered CPR and ventilation video sequences to be distinct in terms of visual content. Therefore, all statistical analyses were conducted separately for the CPR and ventilation scenarios. A p-value < 0.05 was considered statistically significant. Missing data was not imputed. Statistical computations were carried out using IBM SPSS Statistics (Version 29; IBM Inc., Armonk, NY, USA). Sankey plots were generated by using the software “R” (The R Foundation for Statistical Computing. Vienna, Austria).

## Results

In total, 73 medical professionals were recruited, thereof 30 emergency physicians and 43 paramedics. Notably, following a review during the ongoing recruitment process, 12 participants were retrospectively excluded after participating in the study due to non-fulfillment of the inclusion criteria. These individuals were trainees in a paramedic certification course who had not yet completed their final examination. Thus, 30 emergency physicians and 31 EMS staff members were included in the final data analysis (Fig. 2). All questionnaires were completed and returned for statistical analysis. In total, 549 video sequences were evaluated and included for statistical analysis. No adverse events were observed throughout the course of the study. Participants’ characteristics can be found in Table 1.

**Table 1:** Study population. EMS: emergency medical service; eps: emergency physicians.

Characteristics	EMS (*n* = 31)	EPs (*n* = 30)	Total (*n* = 61)	*p*-value
**Age** [years]	30.2 ± 7.6	38.4 ± 5.3	34.2 ± 7.7	< 0.001
**Sex**				0.031
Female [n/%]	4/12.9	11/36.7	15/24.6	
Male [n/%]	27/87.1	19/63.3	46/75.4	
**Experience in emergency service**				0.117
1–2 [n/%]	4/12.9	5/16.7	9/14.8	
3–5 [n/%]	11/35.5	4/13.3	15/24.6	
5–10 [n/%]	9/29.0	7/23.3	16/26.2	
≥ 10 [n/%]	7/22.6	14/46.7	21/34.4	
**Number of passed CPR courses**				< 0.001
0 [n/%]	16/51.6	0/0.0	16/26.2	
1 [n/%]	2/6.5	5/16.7	7/11.5	
2 [n/%]	12/38.7	7/23.3	19/31.1	
3 [n/%]	1/3.2	8/26.7	9/14.8	
≥ 4 [n/%]	0/0.0	10/33.3	10/16.4	

### Primary endpoint

**Fig. 3:**
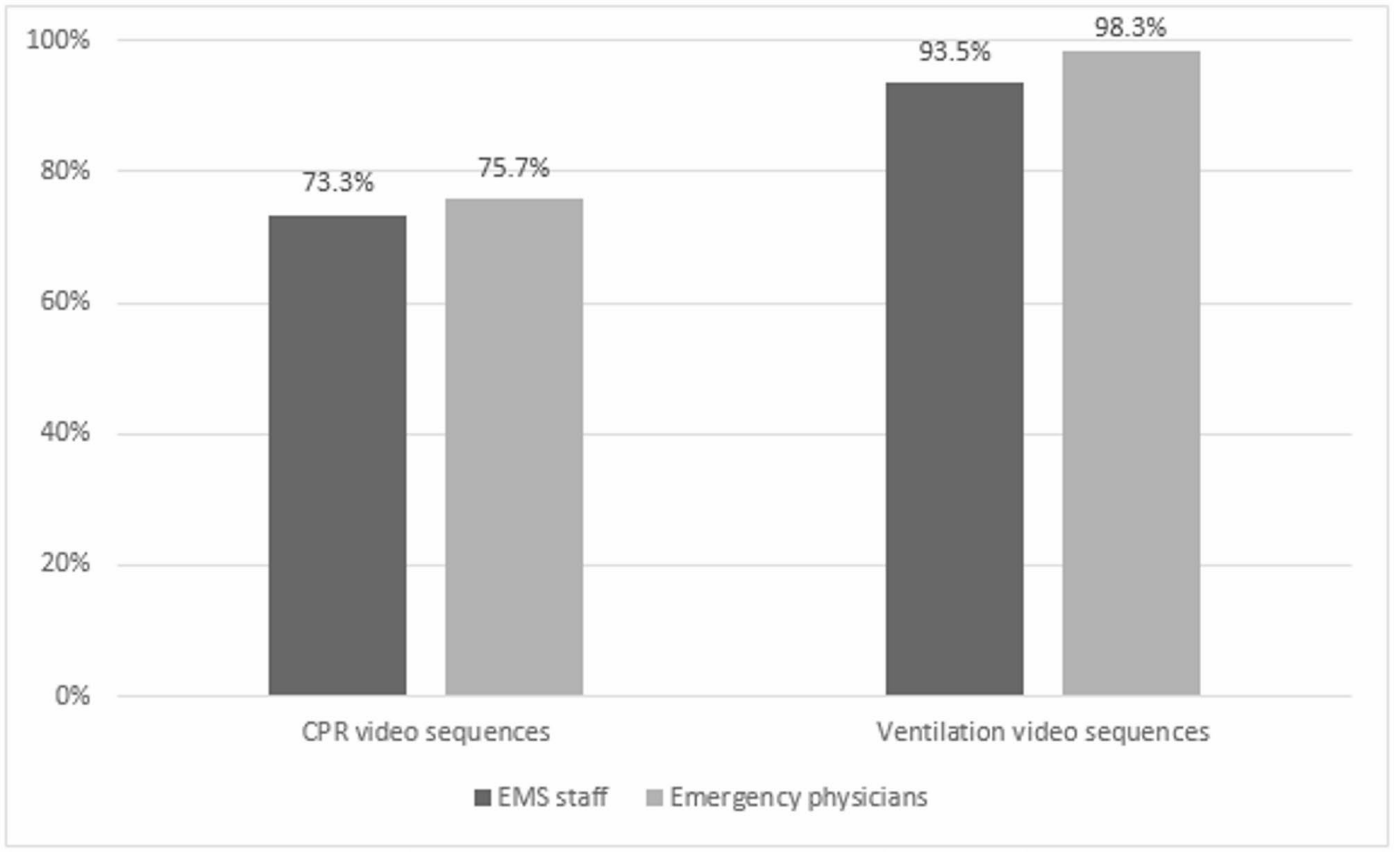
Cumulative frequencies of correct classifications of CPR and ventilation video sequences by professional group, representing the results of the primary endpoint. Results are based on individual video sequences as the unit of analysis, meaning that the figure reflects classifications of video sequences rather than the number of responses from individual participants; no significant differences were observed between groups in CPR scenarios (*p* = 0.213) and ventilation scenarios (*p* = 0.505).

The combined analysis of CPR and ventilation video sequences yielded an overall correct classification rate of 79.2%. In the subsequent analysis focusing exclusively on the CPR video sequences, the correct classification rate was 74.5%. There was no statistically significant differences between EMS personnel and emergency physicians in their ability to classify CPR scenarios (73.3% [159/217] vs. 75.7% [159/210], β = 0.370, SE = 0.297, 95% CI: −0.21 to 0.95, *p* = 0.213) (Fig. 3). Ventilation sequences were correctly classified in 95.9% of cases overall, with classification accuracies of 93.5% (58/62) for EMS personnel and 98.3% (59/60) for emergency physicians, respectively. No statistically significant difference in classification accuracy for ventilation videos was observed between the two professional groups (*β* = 4.50, SE = 6.73, 95% CI: −8.82 to 17.82, *p* = 0.505). Further details can be found as Supplementary Table S1.

### Correct classification related to the presented scenario

**Fig. 4:**
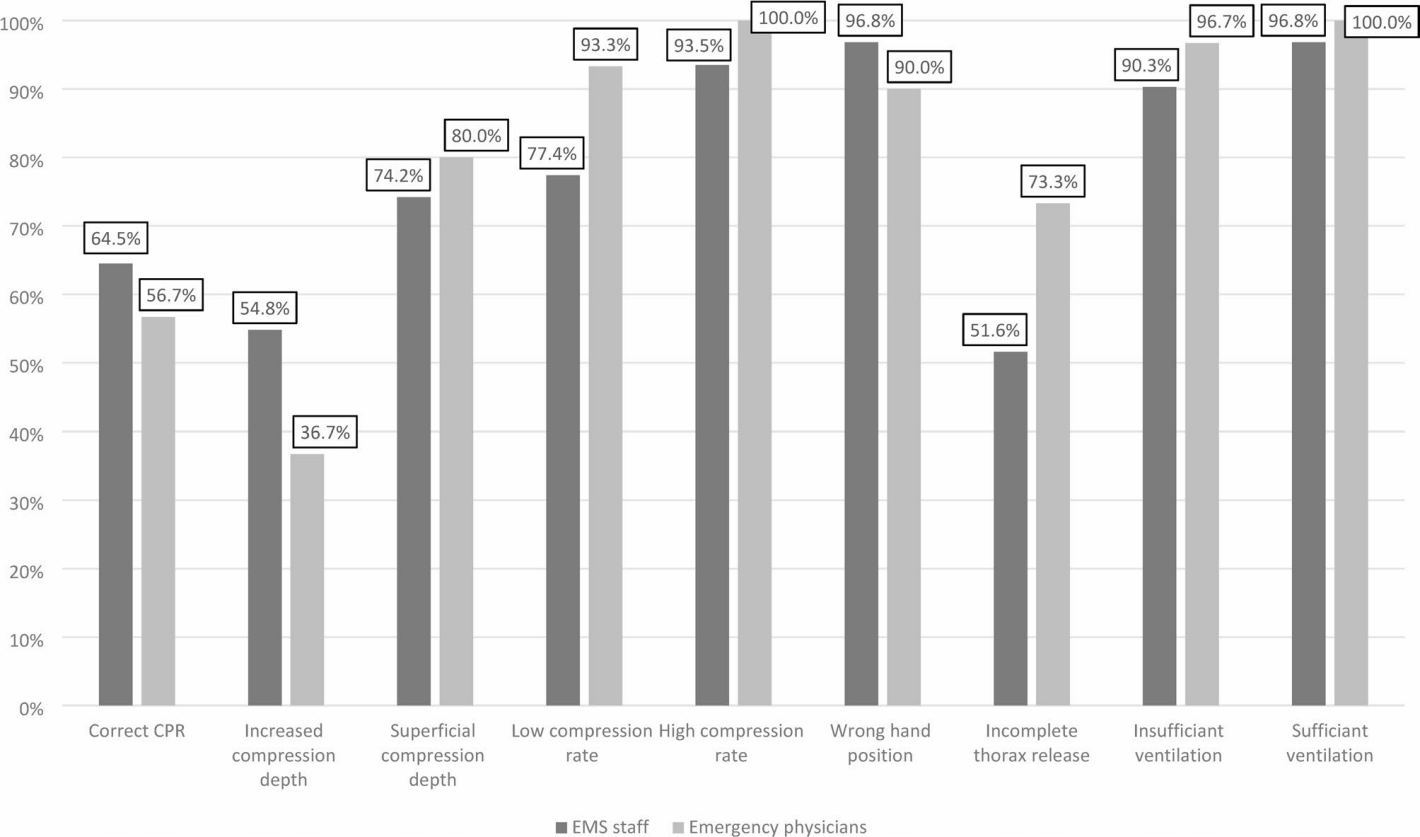
Correct classification of the presented videos, stratified by error type and professional group; classification accuracy varied significantly depending on the specific CPR scenario (*p* < 0.001) but not on the ventilation scenario (*p* = 0.211).

Figure 4 summarizes the correct classification rates for each CPR and ventilation scenario across both professional groups. The specific scenario presented had a significant effect on the classification accuracy in CPR scenarios (*p* < 0.001), whereas no such effect was observed in ventilation scenarios (*p* = 0.211). Further details regarding the influence of the individual CPR and ventilation scenarios are provided as Supplementary Table S2.

### False-positive classification of non-existent errors

**Fig. 5:**
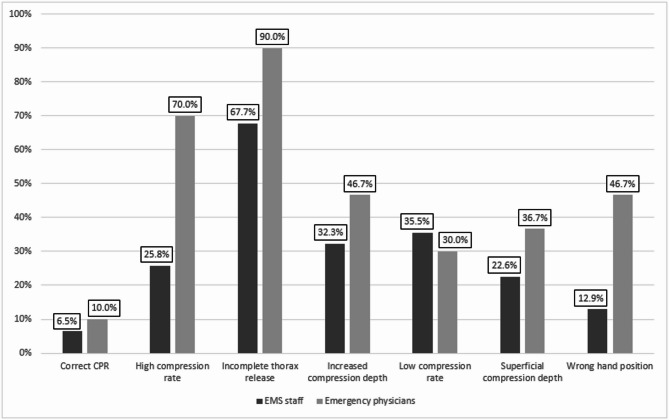
Distribution of false-positive non-existent errors per video sequence, stratified by the presented CPR scenario. Despite instructions that only one error was present per video, participants reported multiple errors in some cases, which were classified as false-positive non-existent errors. A significant effect of the presented CPR scenario on the evaluators’ false-positive classification of non-existent errors was observed (*p* < 0.001).

Despite being informed that each video contained only one specific error (or correct performance), some participants marked non-existent errors in addition to the actually presented scenario (Fig. 5). For CPR, there was a significant effect of the presented CPR scenario on the evaluator’s false-positive indication of non-existent errors (*p* < 0.001) (see Supplementary Table S3).

**Fig. 6:**
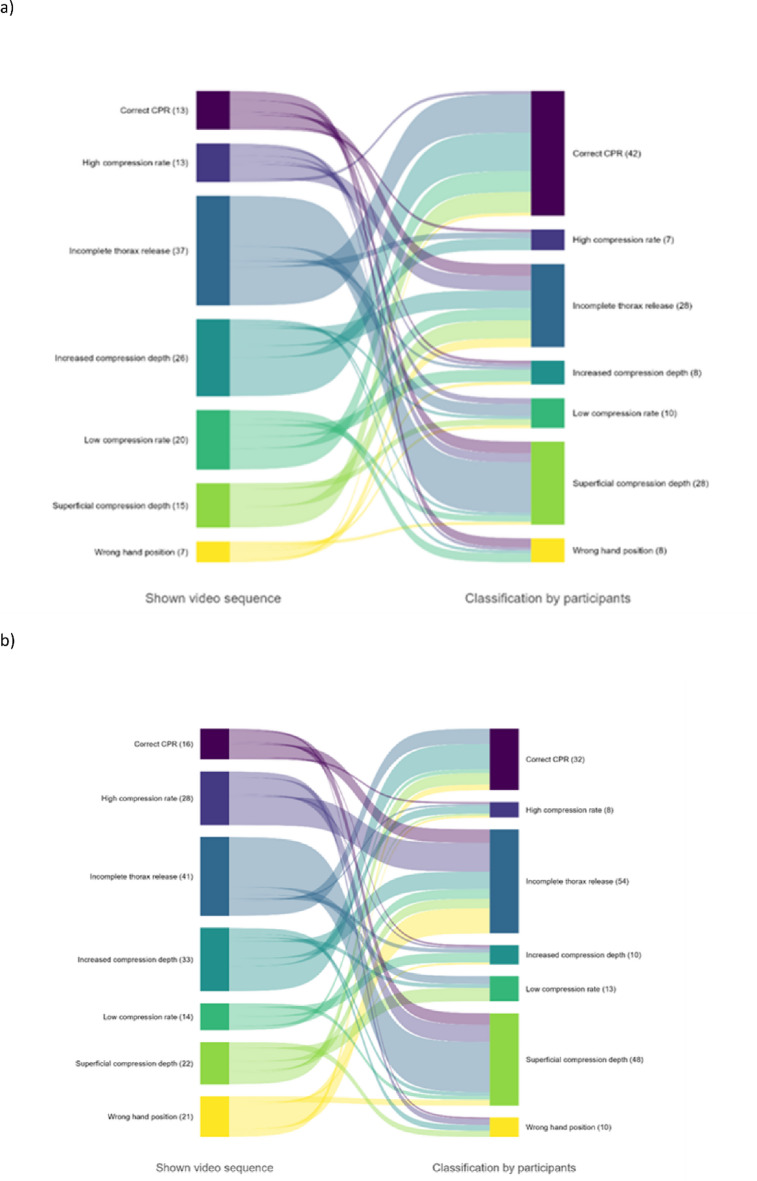
Visualization of misclassifications: the left panel depicts CPR scenarios in which misclassifications occurred. The right panel illustrates the distribution of these misclassifications across error categories, including false-positive non-existent errors. The flows between the panels represent the number of misclassifications transitioning from each CPR scenario to the respective error categories. (**a**) EMS personnel; (**b**) emergency physicians.

Finally, we analyzed the qualitative and quantitative distribution of the false classification depending on the presented video sequences. For each CPR scenario, the frequencies of incorrect classifications were aggregated, including both primary false classifications and instances in which a non-existent error was erroneously indicated (Fig. 6A and B).

The CPR scenario “incomplete thorax release” was most frequently misclassified, or a non-existent error was observed within this scenario by both professional groups: 37 times (37/132 = 28%) by EMS staff and 41 times (41/175 = 23.4%) by emergency physicians (Fig. 6A and B, left column). In addition, the most common misclassification by EMS personnel was the label “correct CPR” (*n* = 42; 42/131 = 32.1%), meaning that a video depicting a CPR scenario with an error was erroneously classified as “correct CPR”. If indicated by emergency physicians, “incomplete thorax release” was most frequently misevaluated (54/175 = 30.9%) (Fig. 6A and B, right column).

### Regression analysis

Two separate linear mixed-effect models were conducted to examine the association between participant characteristics and the correct classification of chest compression and ventilation videos. The predictors included age, gender, professional experience, and number of passed CPR courses. The first analysis included video sequences showing CPR scenarios (*n* = 427). To examine whether the correct classification of CPR video sequences was associated with participants’ profession, age, gender, professional experience, or the number of passed CPR courses, a linear mixed-effects model with normal distribution and identity link function was fitted. The overall model was not statistically significant (F(5, 55) = 0.367, *p* = 0.869), and none of the included predictors showed a significant association with the outcome. Specifically, profession (β = − 0.097, SE = 0.473, *p* = 0.838, 95% CI [–1.046, 0.852]), age (β = − 0.018, SE = 0.032, *p* = 0.580, 95% CI [–0.083, 0.047]), gender (β = 0.448, SE = 0.387, *p* = 0.253, 95% CI [–0.328, 1.224]), professional experience (β = − 0.016, SE = 0.205, *p* = 0.938, 95% CI [–0.426, 0.394]), and the number of passed CPR courses (β = 0.052, SE = 0.131, *p* = 0.691, 95% CI [–0.210, 0.315]) all failed to reach statistical significance. The explained variance of the model was low (marginal R² = 0.032). A comparable model was applied to the analysis of the ventilation video sequences. Again, no statistically significant associations were found between the outcome and any of the included predictors. The model did not reach significance (F(5, 55) = 1.574, *p* = 0.183), and none of the fixed effects yielded significant estimates: profession (β = − 0.085, SE = 0.130, *p* = 0.515, 95% CI [–0.344, 0.175]), age (β = − 0.003, SE = 0.009, *p* = 0.700, 95% CI [–0.021, 0.014]), gender (β = − 0.017, SE = 0.106, *p* = 0.873, 95% CI [–0.229, 0.195]), professional experience (β = − 0.030, SE = 0.056, *p* = 0.599, 95% CI [–0.142, 0.083]), and number of passed CPR courses (β = 0.057, SE = 0.036, *p* = 0.118, 95% CI [–0.015, 0.129]) (see Supplementary Table S4). Although the model explained a slightly larger proportion of variance compared to the CPR model (marginal R² = 0.125), the overall explanatory power remained limited. Collinearity diagnostics revealed a notable correlation between age and professional experience (Pearson *r* = 0.67), with corresponding variance inflation factors of 2.68 (age) and 2.08 (professional experience) and a pronounced negative correlation between their estimated fixed effects (*r* = − 0.67) (see Supplementary Table S5). The tolerance values for gender and number of passed CPR courses were above 0.5, indicating acceptable levels of multicollinearity, while age, profession and professional experience showed values below 0.5. Thus, to assess the potential influence of collinearity between age and professional experience, an additional model excluding age was estimated. The results did not substantially alter the conclusions and are presented in the Supplementary Table S4. Further details including a forest plot are provided as Supplementary Figure S1.

## Discussion

The findings of the present study indicate that the correct classification of CPR and ventilation scenarios within a video sequence does not depend on the evaluator’s profession. “Increased compression depth”, “incomplete chest release” and “correct CPR” were identified less frequently. Individual factors including age, sex, professional experience, and the number of passed CPR courses did not significantly influence the accurate classification of CPR or ventilation video sequences.

Video-assisted CPR can be considered an evolution of the established telephone-based CPR, which has been utilized for over four decades and has undergone numerous modifications^20–31^. Live video communication holds substantial potential to improve emergency interactions between bystanders and dispatch centers. The principal benefits derive from the enhanced situational awareness of the dispatcher, who can directly assess on-scene conditions and make decisions based on visual input. Real-time visual information allows dispatchers to capture relevant details without the need for verbal elicitation, thereby facilitating faster and more targeted intervention. Studies on multimodal saliency demonstrate that audiovisual integration can enhance attention to relevant visual details, especially when visual and auditory cues are strongly aligned​^32^. These findings support the potential of video-assisted dispatcher systems to improve the quality and effectiveness of CPR guidance.

Linderoth et al. demonstrated that the integration of live video to an emergency call is feasible and may enhance the dispatcher’s ability to assess a patient’s condition^9^. In contrast, the initial recognition of cardiac arrest remains challenging when based solely on audio communication, as inherent limitations of telephone-assisted CPR (T-CPR) restrict the dispatcher’s access to relevant visual cues^16,17^. In their simulation studies, Ecker et al. found that a video livestream during cardiopulmonary resuscitation can support the dispatcher’s recognition and correction of typical resuscitation mistakes^10,11^. Specifically, the authors reported non-inferiority of video-assisted CPR compared to telephone-assisted CPR with respect to compression rate and compression depth^10^, and a significantly higher proportion of correct hand positioning under video guidance^11^. These findings are further supported by 2 meta-analyses^18,19^. Lin et al. found a significant improvement in chest compression rate and a trend for correction of the hand position in video-assisted CPR^19^. Similarly, Bielski et al. reported a higher proportion of adequate chest compressions regarding frequency and depth when compared to conventional T-CPR, with a statistically significant improvement in compression rate^19^. Notably, video-assisted CPR was also associated with a significantly increased rate of prehospital return of spontaneous circulation and a 2.5-fold higher likehood of survival to hospital discharge with favorable neurological outcomes. In the same study, incorrect hand positioning was accurately identified in over 90% of cases in both study groups.

Despite high rates of correct classification for individual errors, approximately one fourth of CPR scenarios were misinterpreted, irrespective of the evaluator’s professional background. These results are consistent with results from a previous study by our group, which demonstrated comparable classification accuracy for CPR video sequences (74.5% here, 81.3%^14^). In that earlier study^14^, which was not specifically designed or powered to detect profession-related differences, no significant difference was found, suggesting that the ability to evaluate video-assisted CPR is not profession-dependent. Results of the current study corroborate these results and further demonstrate that the evaluation of ventilation scenarios in video sequences does not significantly differ between professional groups. However, a direct comparison of the two studies must be made with caution. Wetsch et al.^14^ investigated different camera perspectives which may have influenced participants’ evaluations and limit the comparability of findings. Additionally, differences in group composition - particularly regarding age, gender distribution, and the number of passed CPR courses - may have affected participants’ performance in evaluating CPR quality in the present study. In this context, it is noteworthy that a moderate correlation was observed especially between age and professional experience, indicating conceptual overlap between these variables and raising concerns about potential multicollinearity. Further, there was a strong negative correlation between the parameter estimated for age and professional experience. While neither variable demonstrated a statistically significant association with classification accuracy, this statistical pattern may imply that, within the model, older participants performed worse than younger ones at comparable levels of professional experience. However, given the lack of statistical significance and the presence of collinearity between predictors, this interpretation should be treated with caution.

Bolle et al. (2009) investigated the effect of video support using a Nokia N90 3G mobile phone in the context of dispatcher-assisted telephone CPR. The authors concluded that the shown non-superiority of video-assisted CPR was mainly caused by low video quality^33^. Building upon this conclusion, our research group conducted a follow-up study in 2019 to systematically assess the influence of video quality on CPR evaluation. In that study, we examined a range of video resolutions - from 128 × 96 pixels to 1920 × 1080 pixels. Thereby, we did not find a statistically significant effect of video quality on the accuracy of video sequence assessment^15^. In contrast to the findings of Bolle et al., our data suggest that high video quality does not substantially influence the assessment of video-assisted CPR. Despite the generally high video quality in the present study, the recognition of a correct CPR scenario remained relatively low, at 64.5% among EMS personnel and at 56.7% among emergency physicians. This finding underscores a general need for targeted training in the evaluation of video-assisted CPR for dispatchers. Notably, this requirement for training was similarly emphasized in a legal assessment of T-CPR standards dating back to 1987^34^. Regarding misclassification of video sequences, “incomplete chest release” was frequently mistaken for “superficial compression depth” by both professional groups. Interestingly, this commonly confused pair - ‘incomplete chest release’ and ‘superficial compression depth’ - was also reported in a previous study^15^. Both conditions, “incomplete chest release” and “superficial compression depth”, are hallmarked by a reduced amplitude of chest impression. If a compression with incomplete thoracic release is followed by a correct compression, the resultant movement amplitude is noticeably shortened which may lead to its misinterpretation as superficial compression. These observations highlight the inherent difficulty in accurately assessing absolute compression depth via video analysis. In contrast, correct compression rate and hand positioning were evaluated with greater reliability. Therefore, it can be concluded that the visual assessment of compression depth in video recordings poses a greater challenge compared to the evaluation of compression frequency or correct hand placement. Prospectively, technical innovations and the integration of artificial intelligence (AI) could contribute to improve the assessment of CPR in a video stream. Current AI applications predominantly focus on audio analysis, such as automated keyword recognition from callers to facilitate early identification of out-of-hospital cardiac arrest (OHCA)^35^, or AI-driven voice assistants providing guidance to lay rescuers^36^.

## Limitations

The present study is characterized by several limiting factors. The CPR efforts presented in the video sequences may seem artificial. In real life, more than one error may occur simultaneously. Due to the high proportion of error-containing videos, participants may have inferred the intended focus of the study and consequently developed an expectancy bias, leading them to actively search for errors in the presented chest compression videos. Video sequences were recorded with the help of a tripod. Thus, we simulated a second helper holding the smartphone in an optimal position and perfectly still without any instructions. CPR was performed on a training manikin with optimized lighting conditions. Video sequences were evaluated in a controlled environment without distraction. The selected camera angle may limit the ability to judge thoracic recoil. This choice was made to replicate practical conditions based on previous studies but may not represent all possible viewing angles. Furthermore, the assessment of actual video-assisted cardiopulmonary resuscitation necessitates sustained vigilance from the dispatcher for a duration exceeding 10 s. In summary, it can be assumed that the evaluation of video-assisted CPR under real conditions is even more complex.

## Conclusion

Our results indicate that neither professional background nor age significantly influence the ability to identify CPR and ventilation errors in video sequences. Compression depth errors showed the highest rate of misclassification, often accompanied by the selection of non-existent errors. Future research should address how misclassifications - especially of compression-related errors - can be systematically reduced.

## Electronic supplementary material

Below is the link to the electronic supplementary material.

Supplementary Material 1

Supplementary Material 2

Supplementary Material 3

Supplementary Material 4

Supplementary Material 5

Supplementary Material 6

## Data Availability

The datasets generated and analyzed during the current study are available in the RWTH publications repository, https://.publications.rwth-aachen.de/record/999901 and will be published under DOI 10.18154/RWTH-2024-12382 .
