# Supplementary material for: Influence of professional background on assessment of simulated cardiopulmonary resuscitation videos in an observational study
Source: Sci Rep. 2025 Jul 29;15:27648. doi: 10.1038/s41598-025-12306-x (PMC12307580; doi:10.1038/s41598-025-12306-x)
Supplement: Supplementary file 1 — Supplementary Material 1 [file 41598_2025_12306_MOESM1_ESM.pdf]

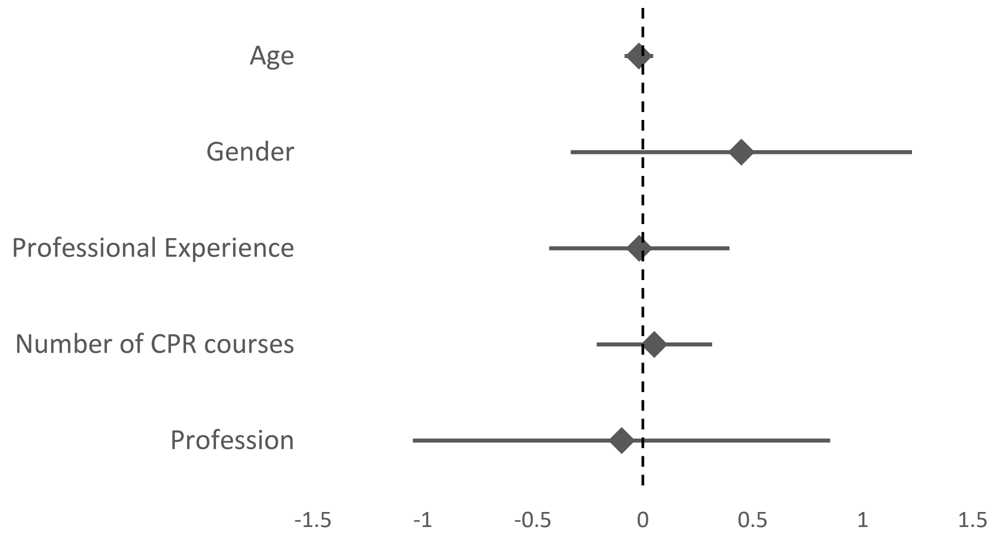

Co-efficients	Lower limit	Upper limit	p-value
-0.018	-0.083	0.047	0.580
0.448	-0.328	1.224	0.253
-0.016	-0.426	0.394	0.938
0.052	-0.210	0.315	0.691
-0.097	-1.046	0.852	0.838

Supplementary Figure S1: Forest plot showing regression coefficients and 95% confidence intervals from the linear mixed-effects model examining associations between participant characteristics and classification accuracy in CPR scenarios.
