# Supplementary material for: Influence of professional background on assessment of simulated cardiopulmonary resuscitation videos in an observational study
Source: Sci Rep. 2025 Jul 29;15:27648. doi: 10.1038/s41598-025-12306-x (PMC12307580; doi:10.1038/s41598-025-12306-x)
Supplement: Supplementary file 2 — Supplementary Material 2 [file 41598_2025_12306_MOESM2_ESM.pdf]

Supplementary Table S1

This document contains the original output from SPSS for a mixed-effects logistic regression model (GENLINMIXED), investigating whether the profession (emergency medical service vs. emergency physician) influences the correct classification of various a) CPR and b) ventilation scenarios. The first analysis includes seven scenarios of CPR performance, while the second analysis covers two ventilation-related scenarios. In both models, repeated measures per participant were considered, as each scenario illustrated a distinct type of performance e rror. Abbreviations and variable codings are explained in the scenario legend below. Fixed effects, confidence intervals, and model statistics are shown. The syntax used for model estimation is shown first, followed by the output tables.

a. Effect of Profession on Correct Error Classification in CPR scenarios

Scenario Legend

Shown CPR scenario 1	Correct CPR
Shown CPR scenario 2	Increased compression depth
Shown CPR scenario 3	Superficial compression depth
Shown CPR scenario 4	Low compression rate
Shown CPR scenario 5	High compression rate
Shown CPR scenario 6	Wrong hand position
Shown CPR scenario 7	Incomplete thorax release
Shown CPR scenario 8	Insufficient ventilation
Shown CPR scenario 9	Sufficient ventilation
Gender 1	Male
Gender 2	Female
Profession 1	Emergency medical service
Profession 2	Emergency physician

Syntax:

```
*Generalized Linear Mixed Models.
GENLINMIXED
  /DATA_STRUCTURE SUBJECTS=ID REPEATED_MEASURES=shown_CPR_scenario COVARIANCE_TYPE=DIAGONAL
  /FIELDS TARGET=correct_classification TRIALS=NONE OFFSET=NONE
  /TARGET_OPTIONS DISTRIBUTION=BINOMIAL LINK=LOGIT
  /FIXED  EFFECTS=profession USE_INTERCEPT=TRUE
  /BUILD_OPTIONS TARGET_CATEGORY_ORDER=ASCENDING INPUTS_CATEGORY_ORDER=ASCENDING
HCONVERGE=0.00000001(RELATIVE) MAX_ITERATIONS=100 CONFIDENCE_LEVEL=95 DF_METHOD=RESIDUAL COVB=MODEL
SCORING=0 SINGULAR=0.000000000001
  /EMMEANS_OPTIONS SCALE=ORIGINAL PADJUST=LSD.
```

Generalized Linear Mixed Models

Case Processing Summary

	N	Percent
Included	427	100,0%
Excluded	0	0,0%
Total	427	100,0%

Model Summary

Target		correct_classification
Probability Distribution		Binomial
Link Function		Logit
Information Criterion	Akaike Corrected	2332,726
	Bayesian	2360,822

Information criteria are based on the -2 log likelihood (2318,457) and are used to compare models. Models with smaller information criterion values fit better.

Data Structure[a]

	Subjects ID	Repeated Measures shown_CPR_scenario	Target correct_classification
Data for First Subject	1	1	yes
	1	2	no
	1	3	no
	1	4	no
	1	5	yes
	1	6	yes
	1	7	no
Total Number of Levels	61	7	

a. Target: correct_classification

Classification Overall Percent Correct = 74,5%[a]

Observed		Predicted no	Predicted yes
no	Count	0	109
	% within Observed	0,0%	100,0%
yes	Count	0	318
	% within Observed	0,0%	100,0%

a. Target: correct_classification

Fixed Effects[a]

Source	F	df1	df2	Sig.
Corrected Model	1,556	1	425	,213
profession	1,556	1	425	,213

Probability distribution: Binomial

Link function: Logit[a]

a. Target: correct_classification

Fixed Effects

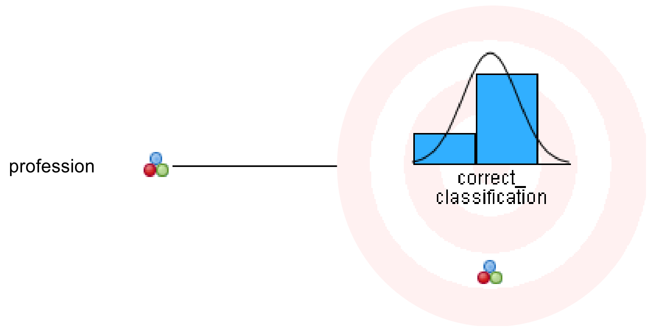

Fixed Coefficients[a]

Model Term	Coefficient	Std. Error	t	Sig.	95% Confidence Interval Lower	95% Confidence Interval Upper	Exp(Coefficient)
Intercept	-2,142	,2255	-9,496	<,001	-2,585	-1,698	,117
profession=1	,370	,2969	1,248	,213	-,213	,954	1,448
profession=2	0[b]	.	.	.	.	.	.

Fixed Coefficients[a]

Model Term	95% Confidence Interval for Exp(Coefficient) Lower	95% Confidence Interval for Exp(Coefficient) Upper
Intercept	,075	,183
profession=1	,808	2,596
profession=2	.	.

Probability distribution: Binomial

Link function: Logit[a]

a. Target: correct_classification

b. This coefficient is set to zero because it is redundant.

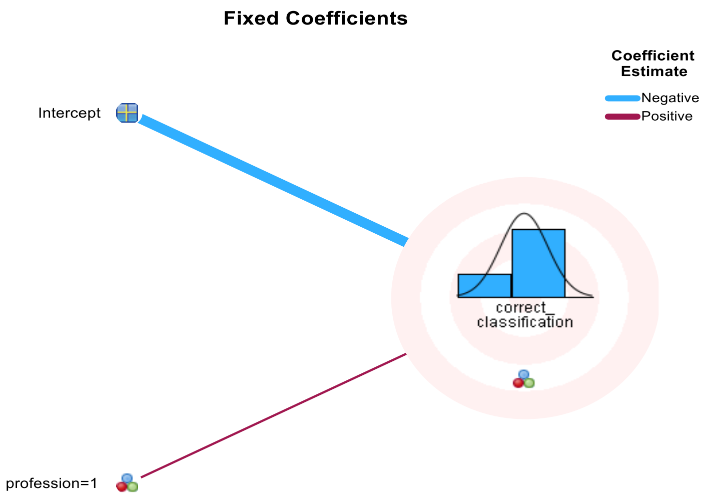

Covariances of Fixed Coefficients[a]

	Intercept	profession=1	profession=2
Intercept	,05086	-,05086	0[b]
profession=1	-,05086	,08813	0[b]
profession=2	0[b]	0[b]	0[b]

Probability distribution: Binomial

Link function: Logit[a]

a. Target: correct_classification

b. One or both coefficients are redundant.

Correlations of Fixed Coefficients[a]

	Intercept	profession=1	profession=2
Intercept	1,000	-,760	.[b]
profession=1	-,760	1,000	.[b]
profession=2	.[b]	.[b]	.[b]

Probability distribution: Binomial
Link function: Logit[a]
a. Target: correct_classification

b. One or both coefficients are redundant.

Covariance Parameters

Covariance Parameters Summary

Covariance Parameters	Residual Effect	7
	Random Effects	0
Design Matrix Columns	Fixed Effects	3
	Random Effects	0[a]
Common Subjects		61

Common subjects are based on the subject specifications for the residual and random effects and are used to chunk the data for better performance.
a. This is the number of columns per common subject.

Residual Effect

Residual Effect	Estimate	Std. Error	Z	Sig.	95% Confidence Interval	
					Lower	Upper
Var(shown_CPR_scenario=1)	2,968	,547	5,429	<,001	2,068	4,258
Var(shown_CPR_scenario=2)	4,074	,754	5,400	<,001	2,834	5,857
Var(shown_CPR_scenario=3)	1,723	,314	5,484	<,001	1,206	2,464
Var(shown_CPR_scenario=4)	1,079	,196	5,497	<,001	,756	1,542
Var(shown_CPR_scenario=5)	,336	,070	4,802	<,001	,223	,505
Var(shown_CPR_scenario=6)	,655	,121	5,402	<,001	,456	,942
Var(shown_CPR_scenario=7)	2,654	,489	5,430	<,001	1,850	3,807

Covariance Structure: Diagonal
Subject Specification: ID

b. Effect of Profession on Correct Error Classification in CPR scenarios

Scenario Legend

Shown CPR scenario 1	Correct CPR
Shown CPR scenario 2	Increased compression depth
Shown CPR scenario 3	Superficial compression depth
Shown CPR scenario 4	Low compression rate
Shown CPR scenario 5	High compression rate
Shown CPR scenario 6	Wrong hand position
Shown CPR scenario 7	Incomplete thorax release
Shown CPR scenario 8	Insufficient ventilation
Shown CPR scenario 9	Sufficient ventilation
Gender 1	Male
Gender 2	Female
Profession 1	Emergency medical service
Profession 2	Emergency physician

```
*Generalized Linear Mixed Models.
GENLINMIXED
  /DATA_STRUCTURE SUBJECTS=ID REPEATED_MEASURES=shown_CPR_scenario
COVARIANCE_TYPE=DIAGONAL
  /FIELDS TARGET=correct_classification TRIALS=NONE OFFSET=NONE
  /TARGET_OPTIONS DISTRIBUTION=BINOMIAL LINK=LOGIT
  /FIXED  EFFECTS=profession USE_INTERCEPT=TRUE
  /BUILD_OPTIONS TARGET_CATEGORY_ORDER=ASCENDING INPUTS_CATEGORY_ORDER=ASCENDING
HCONVERGE=0.00000001(RELATIVE) MAX_ITERATIONS=100 CONFIDENCE_LEVEL=95 DF_METHOD=RESIDUAL
COVB=MODEL SCORING=0 SINGULAR=0.000000000001
  /EMMEANS_OPTIONS SCALE=ORIGINAL PADJUST=LSD.
```

Generalized Linear Mixed Models

Warnings

glmm: The maximum number of iterations was reached but convergence was not achieved. Output for the last iteration is displayed. The procedure continues despite this warning. Subsequent results produced are based on the last iteration. Validity of the model fit is uncertain.

Case Processing Summary

	N	Percent
Included	122	100,0%
Excluded	0	0,0%
Total	122	100,0%

Model Summary

Target		correct_classification
Probability Distribution		Binomial
Link Function		Logit
Information Criterion	Akaike Corrected	1219,126
	Bayesian	1224,599

Information criteria are based on the -2 log likelihood (1215,024) and are used to compare models. Models with smaller information criterion values fit better.

Data Structure[a]

	Subjects<br>ID	Repeated Measures<br>shown_CPR_scenario	Target<br>correct_classification
Data for First Subject	1	8	yes
	1	9	yes
Total Number of Levels	61	2	

a. Target: correct_classification

Classification
Overall Percent Correct = 95,9%[a]

Observed		Predicted no	Predicted yes
no	Count	0	5
	% within Observed	0,0%	100,0%
yes	Count	0	117
	% within Observed	0,0%	100,0%

a. Target: correct_classification

Fixed Effects[a]

Source	F	df1	df2	Sig.
Corrected Model	,447	1	120	,505
profession	,447	1	120	,505

Probability distribution: Binomial
Link function: Logit[a]
a. Target: correct_classification

Fixed Effects

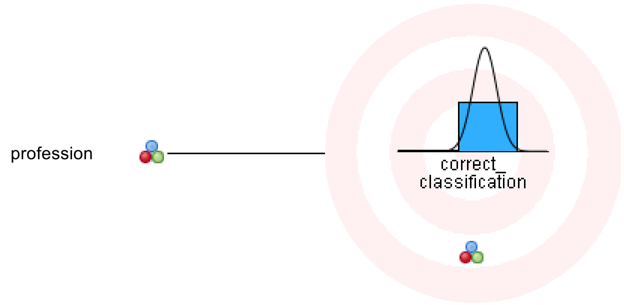

Fixed Coefficients[a]

Model Term	Coefficient	Std. Error	t	Sig.	95% Confidence Interval Lower	95% Confidence Interval Upper	Exp(Coefficient)
Intercept	-7,878	6,6898	-1,178	,241	-21,124	5,367	,000
profession=1	4,500	6,7281	,669	,505	-8,821	17,822	90,047
profession=2	0[b]	.	.	.	.	.	.

Fixed Coefficients[a]

Model Term	95% Confidence Interval for Exp(Coefficient) Lower	95% Confidence Interval for Exp(Coefficient) Upper
Intercept	6,700E-10	214,245
profession=1	,000	54931860,011
profession=2	.	.

Probability distribution: Binomial
Link function: Logit[a]

a. Target: correct_classification

b. This coefficient is set to zero because it is redundant.

Fixed Coefficients

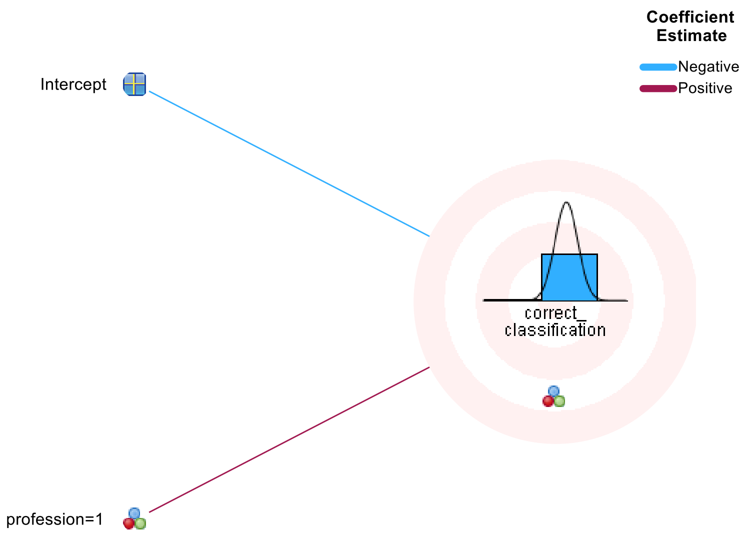

Covariances of Fixed Coefficients[a]

	Intercept	profession=1	profession=2
Intercept	44,75403	-44,75403	0[b]
profession=1	-44,75403	45,26800	0[b]
profession=2	0[b]	0[b]	0[b]

Probability distribution: Binomial
Link function: Logit[a]

a. Target: correct_classification

b. One or both coefficients are redundant.

Correlations of Fixed Coefficients[a]

	Intercept	profession=1	profession=2
Intercept	1,000	-,994	.[b]
profession=1	-,994	1,000	.[b]
profession=2	.[b]	.[b]	.[b]

Probability distribution: Binomial

Link function: Logit[a]

a. Target: correct_classification

b. One or both coefficients are redundant.

Covariance Parameters

Covariance Parameters Summary

Covariance Parameters	Residual Effect	2
	Random Effects	0
Design Matrix Columns	Fixed Effects	3
	Random Effects	0[a]
Common Subjects		61

Common subjects are based on the subject specifications for the residual and random effects and are used to chunk the data for better performance.

a. This is the number of columns per common subject.

Residual Effect

Residual Effect	Estimate	Std. Error	Z	Sig.	95% Confidence Interval	
					Lower	Upper
Var(shown_CPR_scenario=8)	44,737	8,103	5,521	<,001	31,369	63,802
Var(shown_CPR_scenario=9)	,514	,095	5,431	<,001	,358	,738

Covariance Structure: Diagonal
Subject Specification: ID
