# Supplementary material for: Influence of professional background on assessment of simulated cardiopulmonary resuscitation videos in an observational study
Source: Sci Rep. 2025 Jul 29;15:27648. doi: 10.1038/s41598-025-12306-x (PMC12307580; doi:10.1038/s41598-025-12306-x)
Supplement: Supplementary file 3 — Supplementary Material 3 [file 41598_2025_12306_MOESM3_ESM.pdf]

Supplementary Table S2

This document contains the original SPSS output from a mixed-effects logistic regression model (GENLINMIXED), examining whether the type of error presented in a video sequence - either a) a CPR scenario or b) a ventilation scenario - affects the accuracy of error classification by participants. The first analysis includes seven CPR-related scenarios, while the second focuses on two scenarios involving ventilation. In both models, repeated measures per participant were accounted, as each scenario represented a distinct type of performance error. The SPSS syntax used for model estimation is presented first, followed by the corresponding output tables. All abbreviations and variable codings are explained in the scenario legend below. Reported results include fixed effects, confidence intervals, and model fit statistics.

a. Effect of CPR Error Type on Correct Classification Accuracy

Scenario Legend

Shown CPR scenario 1	Correct CPR
Shown CPR scenario 2	Increased compression depth
Shown CPR scenario 3	Superficial compression depth
Shown CPR scenario 4	Low compression rate
Shown CPR scenario 5	High compression rate
Shown CPR scenario 6	Wrong hand position
Shown CPR scenario 7	Incomplete thorax release
Gender 1	Male
Gender 2	Female
Profession 1	Emergency medical service
Profession 2	Emergency physician

Syntax:

```
*Generalized Linear Mixed Models.
GENLINMIXED
  /DATA_STRUCTURE SUBJECTS=ID REPEATED_MEASURES=shown_CPR_scenario
COVARIANCE_TYPE=DIAGONAL
  /FIELDS TARGET=correct_classification TRIALS=NONE OFFSET=NONE
  /TARGET_OPTIONS DISTRIBUTION=BINOMIAL LINK=LOGIT
  /FIXED  EFFECTS=shown_CPR_scenario USE_INTERCEPT=TRUE
  /BUILD_OPTIONS TARGET_CATEGORY_ORDER=ASCENDING INPUTS_CATEGORY_ORDER=ASCENDING
HCONVERGE=0.00000001(RELATIVE) MAX_ITERATIONS=100 CONFIDENCE_LEVEL=95 DF_METHOD=RESIDUAL
COVB=MODEL SCORING=0 SINGULAR=0.000000000001
  /EMMEANS_OPTIONS SCALE=ORIGINAL PADJUST=LSD.
```

Generalized Linear Mixed Models

Case Processing Summary

	N	Percent
Included	427	100,0%
Excluded	0	0,0%
Total	427	100,0%

Model Summary

Target		correct_classification
Probability Distribution		Binomial
Link Function		Logit
Information Criterion	Akaike Corrected	2101,325
	Bayesian	2129,335

Information criteria are based on the -2 log likelihood (2087,053) and are used to compare models. Models with smaller information criterion values fit better.

Data Structure[a]

	Subjects<br>ID	Repeated Measures<br>shown_CPR_scenario	Target<br>correct_classification
Data for First Subject	1	1	yes
	1	2	no
	1	3	no
	1	4	no
	1	5	yes
	1	6	yes
	1	7	no
Total Number of Levels	61	7	

a. Target: correct_classification

Classification
Overall Percent Correct = 75,6%[a]

Observed		Predicted no	Predicted yes
no	Count	33	76
	% within Observed	30,3%	69,7%
yes	Count	28	290
	% within Observed	8,8%	91,2%

a. Target: correct_classification

Fixed Effects[a]

Source	F	df1	df2	Sig.
Corrected Model	8,513	6	420	<,001
shown_CPR_scenario	8,513	6	420	<,001

Probability distribution: Binomial
Link function: Logit
a

a. Target: correct_classification

Fixed Effects

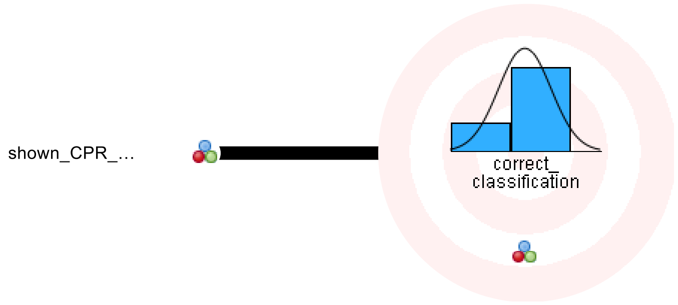

Fixed Coefficients[a]

Model Term	Coefficient	Std. Error	t	Sig.	95% Confidence Interval Lower	95% Confidence Interval Upper
Intercept	-,502	,2664	-1,885	,060	-1,026	,022
shown_CPR_scenario=1	,069	,3752	,184	,854	-,668	,807
shown_CPR_scenario=2	,666	,3716	1,793	,074	-,064	1,397
shown_CPR_scenario=3	-,709	,4065	-1,744	,082	-1,508	,090
shown_CPR_scenario=4	-1,252	,4511	-2,775	,006	-2,139	-,365
shown_CPR_scenario=5	-2,882	,7723	-3,732	<,001	-4,400	-1,364
shown_CPR_scenario=6	-2,155	,5856	-3,679	<,001	-3,306	-1,004
shown_CPR_scenario=7	0[b]	.	.	.	.	.

Fixed Coefficients[a]

Model Term	Exp(Coefficient)	95% Confidence Interval for Exp(Coefficient) Lower	95% Confidence Interval for Exp(Coefficient) Upper
Intercept	,605	,359	1,022
shown_CPR_scenario=1	1,072	,513	2,241
shown_CPR_scenario=2	1,947	,938	4,042
shown_CPR_scenario=3	,492	,221	1,094
shown_CPR_scenario=4	,286	,118	,694
shown_CPR_scenario=5	,056	,012	,256
shown_CPR_scenario=6	,116	,037	,367
shown_CPR_scenario=7	.	.	.

Probability distribution: Binomial

Link function: Logit[a]

a. Target: correct_classification

b. This coefficient is set to zero because it is redundant.

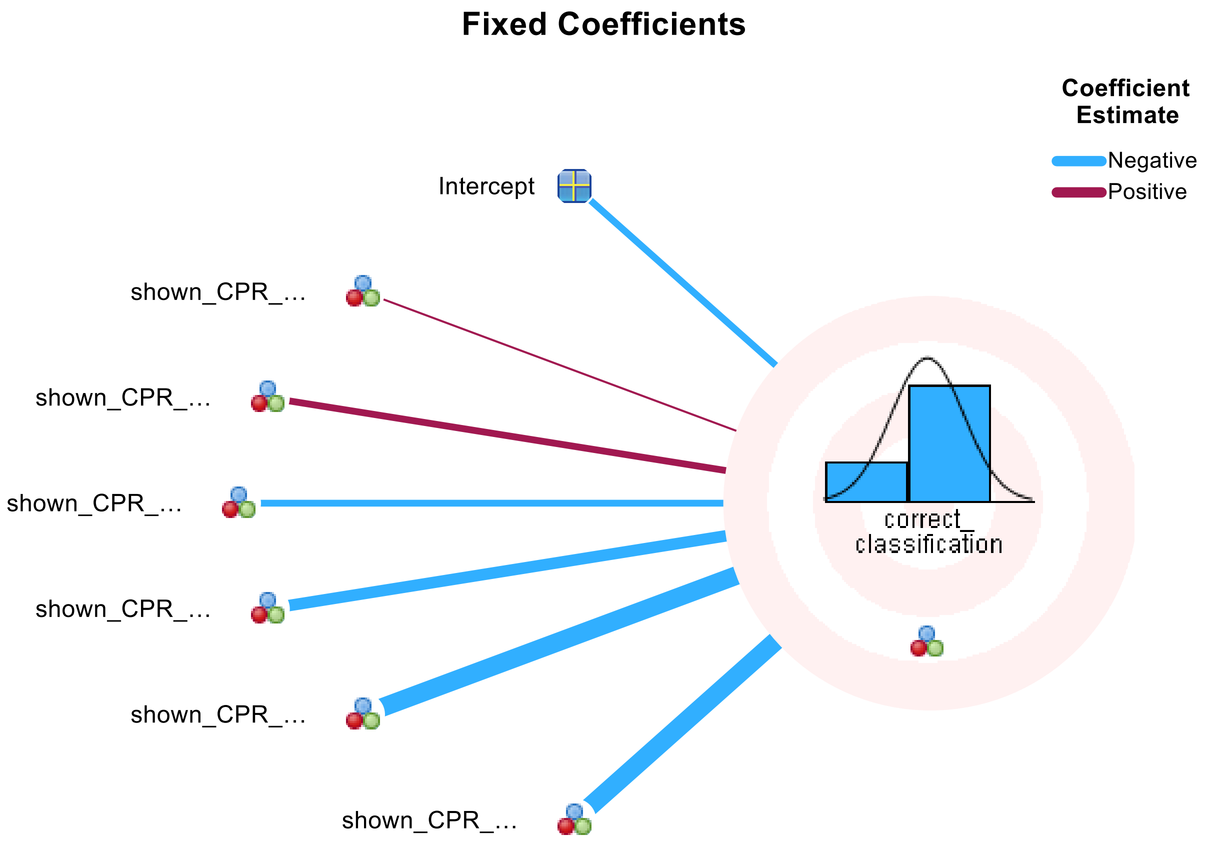
Fixed Coefficients
Coefficient Estimate
Negative
Positive
Intercept
shown_CPR_...
shown_CPR_...
shown_CPR_...
shown_CPR_...
shown_CPR_...
shown_CPR_...
correct_
classification

Covariances of Fixed Coefficients[a]

	Intercept	shown_CPR_scenario=1	shown_CPR_scenario=2	shown_CPR_scenario=3	shown_CPR_scenario=4
Intercept	,07096	-,07096	-,07096	-,07096	-,07096
shown_CPR_scenario=1	-,07096	,14080	,07096	,07096	,07096
shown_CPR_scenario=2	-,07096	,07096	,13807	,07096	,07096
shown_CPR_scenario=3	-,07096	,07096	,07096	,16521	,07096
shown_CPR_scenario=4	-,07096	,07096	,07096	,07096	,20347
shown_CPR_scenario=5	-,07096	,07096	,07096	,07096	,07096
shown_CPR_scenario=6	-,07096	,07096	,07096	,07096	,07096
shown_CPR_scenario=7	0[b]	0[b]	0[b]	0[b]	0[b]

Covariances of Fixed Coefficients[a]

	shown_CPR_scenario=5	shown_CPR_scenario=6	shown_CPR_scenario=7
Intercept	-,07096	-,07096	0[b]
shown_CPR_scenario=1	,07096	,07096	0[b]
shown_CPR_scenario=2	,07096	,07096	0[b]
shown_CPR_scenario=3	,07096	,07096	0[b]
shown_CPR_scenario=4	,07096	,07096	0[b]
shown_CPR_scenario=5	,59652	,07096	0[b]
shown_CPR_scenario=6	,07096	,34296	0[b]
shown_CPR_scenario=7	0[b]	0[b]	0[b]

Probability distribution: Binomial
Link function: Logit[a]

a. Target: correct_classification

b. One or both coefficients are redundant.

Correlations of Fixed Coefficients[a]

	Intercept	shown_CPR_scenario=1	shown_CPR_scenario=2	shown_CPR_scenario=3	shown_CPR_scenario=4
Intercept	1,000	-,710	-,717	-,655	-,591
shown_CPR_scenario=1	-,710	1,000	,509	,465	,419
shown_CPR_scenario=2	-,717	,509	1,000	,470	,423
shown_CPR_scenario=3	-,655	,465	,470	1,000	,387
shown_CPR_scenario=4	-,591	,419	,423	,387	1,000
shown_CPR_scenario=5	-,345	,245	,247	,226	,204
shown_CPR_scenario=6	-,455	,323	,326	,298	,269
shown_CPR_scenario=7	.[b]	.[b]	.[b]	.[b]	.[b]

Correlations of Fixed Coefficients[a]

	shown_CPR_scenario=5	shown_CPR_scenario=6	shown_CPR_scenario=7
Intercept	-,345	-,455	.[b]
shown_CPR_scenario=1	,245	,323	.[b]
shown_CPR_scenario=2	,247	,326	.[b]
shown_CPR_scenario=3	,226	,298	.[b]
shown_CPR_scenario=4	,204	,269	.[b]
shown_CPR_scenario=5	1,000	,157	.[b]
shown_CPR_scenario=6	,157	1,000	.[b]
shown_CPR_scenario=7	.[b]	.[b]	.[b]

Probability distribution: Binomial
Link function: Logit[a]

a. Target: correct_classification

b. One or both coefficients are redundant.

Covariance Parameters

Covariance Parameters Summary

Covariance Parameters	Residual Effect	7
	Random Effects	0
Design Matrix Columns	Fixed Effects	8
	Random Effects	0[a]
Common Subjects		61

Common subjects are based on the subject specifications for the residual and random effects and are used to chunk the data for better performance.

a. This is the number of columns per common subject.

Residual Effect

Residual Effect	Estimate	Std. Error	Z	Sig.	95% Confidence Interval Lower	Upper
Var(shown_CPR_scenario=1)	1,017	,186	5,477	<,001	,711	1,454
Var(shown_CPR_scenario=2)	1,017	,186	5,477	<,001	,711	1,454
Var(shown_CPR_scenario=3)	1,017	,186	5,477	<,001	,711	1,454
Var(shown_CPR_scenario=4)	1,017	,186	5,477	<,001	,711	1,454
Var(shown_CPR_scenario=5)	1,017	,186	5,477	<,001	,711	1,454
Var(shown_CPR_scenario=6)	1,017	,186	5,477	<,001	,711	1,454
Var(shown_CPR_scenario=7)	1,017	,186	5,477	<,001	,711	1,454

Covariance Structure: Diagonal
Subject Specification: ID

Estimated Marginal Means for Top Significant Fixed Effects

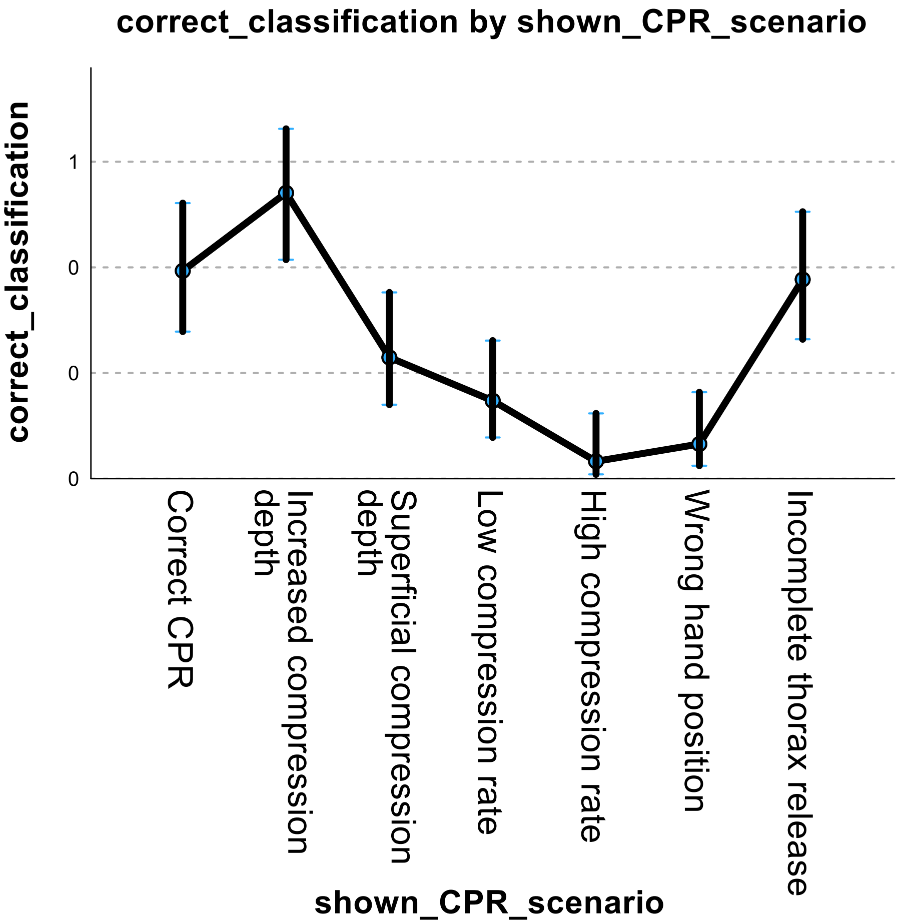

Effect of Ventilation Error Type on Correct Classification Accuracy

Scenario Legend

Shown CPR scenario 1	Correct CPR
Shown CPR scenario 2	Increased compression depth
Shown CPR scenario 3	Superficial compression depth
Shown CPR scenario 4	Low compression rate
Shown CPR scenario 5	High compression rate
Shown CPR scenario 6	Wrong hand position
Shown CPR scenario 7	Incomplete thorax release
Shown CPR scenario 8	Insufficient ventilation
Shown CPR scenario 9	Sufficient ventilation
Gender 1	Male
Gender 2	Female
Profession 1	Emergency medical service
Profession 2	Emergency physician

Syntax:

```
*Generalized Linear Mixed Models.
GENLINMIXED
  /DATA_STRUCTURE SUBJECTS=ID REPEATED_MEASURES=shown_CPR_scenario COVARIANCE_TYPE=DIAGONAL
  /FIELDS TARGET=correct_classification TRIALS=NONE OFFSET=NONE
  /TARGET_OPTIONS DISTRIBUTION=BINOMIAL LINK=LOGIT
  /FIXED  EFFECTS=shown_CPR_scenario USE_INTERCEPT=TRUE
  /BUILD_OPTIONS TARGET_CATEGORY_ORDER=ASCENDING INPUTS_CATEGORY_ORDER=ASCENDING
HCONVERGE=0.00000001(RELATIVE) MAX_ITERATIONS=100 CONFIDENCE_LEVEL=95 DF_METHOD=RESIDUAL
COVB=MODEL SCORING=0 SINGULAR=0.000000000001
  /EMMEANS TABLES=shown_CPR_scenario CONTRAST=NONE
  /EMMEANS_OPTIONS SCALE=ORIGINAL PADJUST=LSD.
```

Generalized Linear Mixed Models

Case Processing Summary

	N	Percent
Included	122	100,0%
Excluded	0	0,0%
Total	122	100,0%

Model Summary

Target		correct_classification
Probability Distribution		Binomial
Link Function		Logit
Information Criterion	Akaike Corrected	770,041
	Bayesian	775,514

Information criteria are based on the -2 log likelihood (765,939) and are used to compare models. Models with smaller information criterion values fit better.

Data Structure[a]

	Subjects<br>ID	Repeated Measures<br>shown_CPR_scenario	Target<br>correct_classification
Data for First Subject	1	8	yes
	1	9	yes
Total Number of Levels	61	2	

a. Target: correct_classification

Classification
Overall Percent Correct = 95,9%[a]

Observed		Predicted no	Predicted yes
no	Count	0	5
	% within Observed	0,0%	100,0%
yes	Count	0	117
	% within Observed	0,0%	100,0%

a. Target: correct_classification

Fixed Effects[a]

Source	F	df1	df2	Sig.
Corrected Model	1,583	1	120	,211
shown_CPR_scenario	1,583	1	120	,211

Probability distribution: Binomial

Link function: Logit[a]

a. Target: correct_classification

Fixed Effects

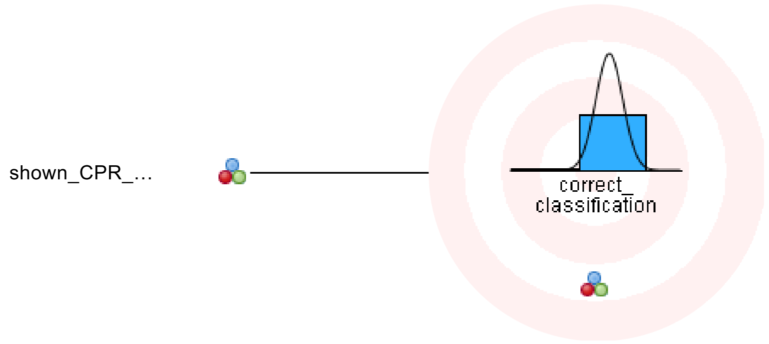

Fixed Coefficients[a]

Model Term	Coefficient	Std. Error	t	Sig.	95% Confidence Interval	
					Lower	Upper
Intercept	-4,094	1,0167	-4,027	<,001	-6,107	-2,081
shown_CPR_scenario =8	1,438	1,1426	1,258	,211	-,825	3,700
shown_CPR_scenario =9	0[b]	.	.	.	.	.

Fixed Coefficients[a]

Model Term	Exp(Coefficient)	95% Confidence Interval for Exp(Coefficient)	
		Lower	Upper
Intercept	,017	,002	,125
shown_CPR_scenario =8	4,211	,438	40,444
shown_CPR_scenario =9	.	.	.

Probability distribution: Binomial
Link function: Logit[a]

a. Target: correct_classification

b. This coefficient is set to zero because it is redundant.

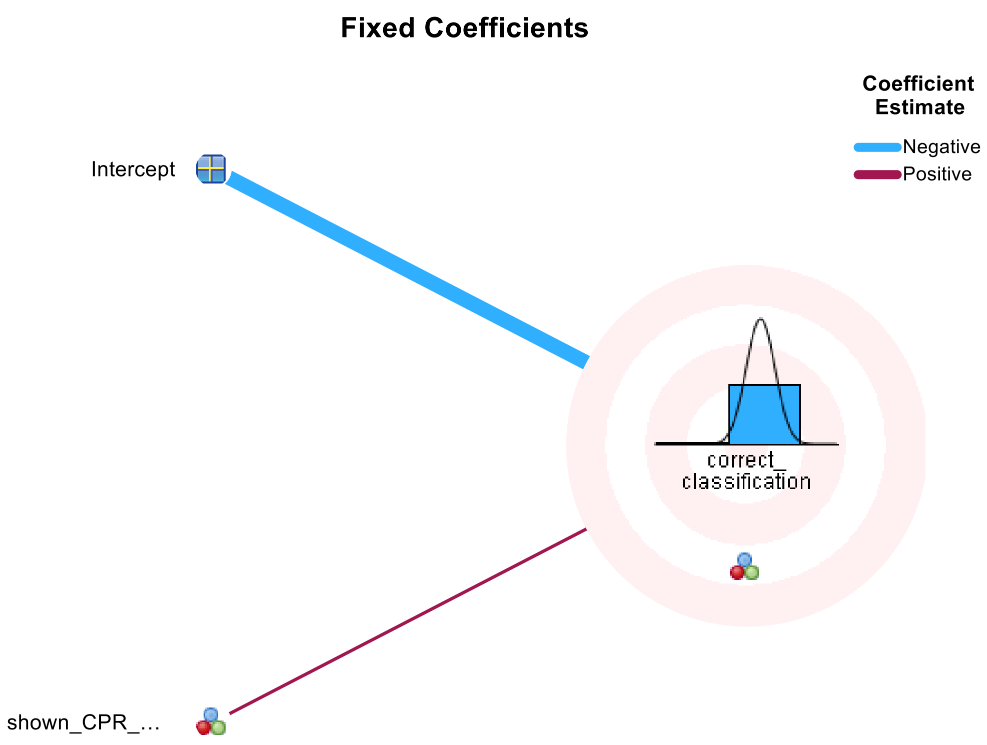

Fixed Coefficients
Coefficient Estimate
Negative
Positive
Intercept
correct_ classification
shown_CPR_...

Covariances of Fixed Coefficients[a]

	Intercept	shown_CPR_scenario=8	shown_CPR_scenario=9
Intercept	1,03361	-1,03361	0[b]
shown_CPR_scenario=8	-1,03361	1,30561	0[b]
shown_CPR_scenario=9	0[b]	0[b]	0[b]

Probability distribution: Binomial
Link function: Logit[a]
a. Target: correct_classification

b. One or both coefficients are redundant.

Correlations of Fixed Coefficients[a]

	Intercept	shown_CPR_scenario=8	shown_CPR_scenario=9
Intercept	1,000	-,890	.[b]
shown_CPR_scenario=8	-,890	1,000	.[b]
shown_CPR_scenario=9	.[b]	.[b]	.[b]

Probability distribution: Binomial
Link function: Logit[a]
a. Target: correct_classification

b. One or both coefficients are redundant.

Covariance Parameters

Covariance Parameters Summary

Covariance Parameters	Residual Effect	2
	Random Effects	0
Design Matrix Columns	Fixed Effects	3
	Random Effects	0[a]
Common Subjects		61

Common subjects are based on the subject specifications for the residual and random effects and are used to chunk the data for better performance.

a. This is the number of columns per common subject.

Residual Effect

Residual Effect	Estimate	Std. Error	Z	Sig.	95% Confidence Interval Lower	95% Confidence Interval Upper
Var(shown_CPR_scenario=8)	1,017	,186	5,477	<,001	,711	1,454
Var(shown_CPR_scenario=9)	1,017	,186	5,477	<,001	,711	1,454

Covariance Structure: Diagonal

Subject Specification: ID

Estimated Means: Grand Mean

Estimates[a]

Mean	Std. Error	95% Confidence Interval Lower	95% Confidence Interval Upper
,033	,018	,011	,096

a. Target: correct_classification

Estimated Means: shown_CPR_scenario

Estimates

shown_CPR_scenario	Mean	Std. Error	95% Confidence Interval Lower	95% Confidence Interval Upper
insufficient ventilation	,066	,032	,024	,165
correct ventilation	,016	,016	,002	,111

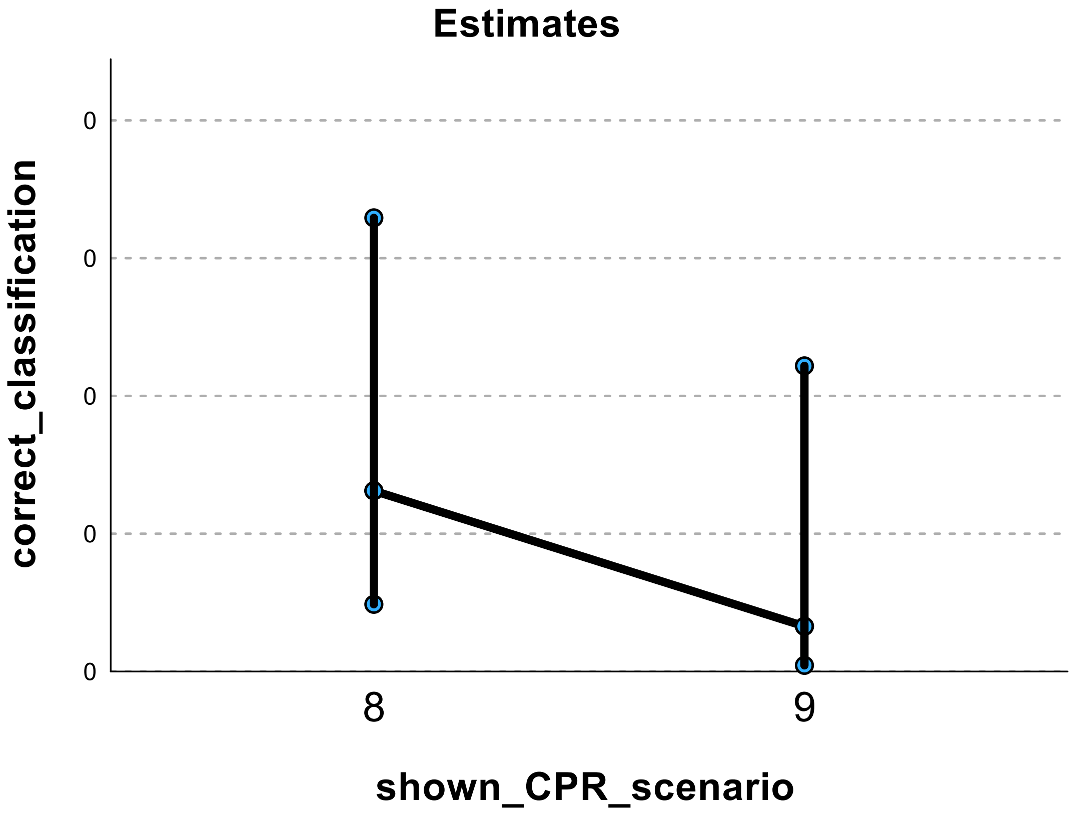

Estimates
0
0
0
0
0
correct_classification
8
9
shown_CPR_scenario
