# Supplementary material for: Influence of professional background on assessment of simulated cardiopulmonary resuscitation videos in an observational study
Source: Sci Rep. 2025 Jul 29;15:27648. doi: 10.1038/s41598-025-12306-x (PMC12307580; doi:10.1038/s41598-025-12306-x)

Supplementary Table S3

This document contains the original SPSS output from a mixed-effects logistic regression model (GENLINMIXED), examining whether the type of CPR performance presented in a video sequence affects the false positive indication of non-existent errors. The analysis includes seven CPR-related scenarios. To account for within-subject correlation, repeated measures per participant were included, as each scenario involved a unique error type. The SPSS syntax used for model estimation is presented first, followed by the corresponding output tables. All abbreviations and variable codings are explained in the scenario legend below. Reported results include fixed effects, confidence intervals, and model fit statistics.

a. Effect of the Presented Error on false-positive indication of non-existent errors

Scenario Legend

Shown CPR scenario 1	Correct CPR
Shown CPR scenario 2	Increased compression depth
Shown CPR scenario 3	Superficial compression depth
Shown CPR scenario 4	Low compression rate
Shown CPR scenario 5	High compression rate
Shown CPR scenario 6	Wrong hand position
Shown CPR scenario 7	Incomplete thorax release
Shown CPR scenario 8	Insufficient ventilation
Shown CPR scenario 9	Sufficient ventilation
Gender 1	Male
Gender 2	Female
Profession 1	Emergency medical service
Profession 2	Emergency physician

Syntax:

```
*Generalized Linear Mixed Models.
GENLINMIXED
  /DATA_STRUCTURE SUBJECTS=ID
  /FIELDS TARGET=number_additional_errors_identified TRIALS=NONE OFFSET=NONE
  /TARGET_OPTIONS DISTRIBUTION=NORMAL LINK=IDENTITY
  /FIXED  EFFECTS=shown_CPR_scenario USE_INTERCEPT=TRUE
  /BUILD_OPTIONS TARGET_CATEGORY_ORDER=ASCENDING INPUTS_CATEGORY_ORDER=ASCENDING
HCONVERGE=0.00000001(RELATIVE) MAX_ITERATIONS=100 CONFIDENCE_LEVEL=95 DF_METHOD=RESIDUAL
COVB=MODEL SCORING=0 SINGULAR=0.000000000001
  /EMMEANS_OPTIONS SCALE=ORIGINAL PADJUST=LSD.
```

Generalized Linear Mixed Models

Warnings

Data Structure: One or more subject fields were specified but not actually used in the analysis.

Case Processing Summary

	N	Percent
Included	427	100,0%
Excluded	0	0,0%
Total	427	100,0%

Model Summary

Target		number_additional_errors_identified
Probability Distribution		Normal
Link Function		Identity
Information Criterion	Akaike Corrected	826,217
	Bayesian	830,248

Information criteria are based on the -2 log likelihood (824,207) and are used to compare models. Models with smaller information criterion values fit better.

Coefficients of Determination

Pseudo-R Square Measures	Marginal	,170
	Conditional	,170

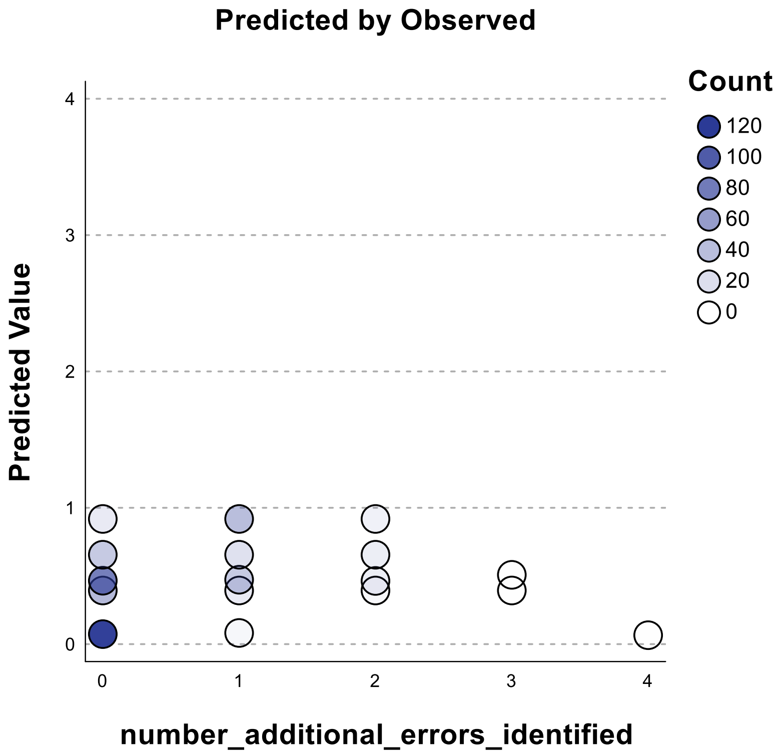

Fixed Effects[a]

Source	F	df1	df2	Sig.
Corrected Model	14,380	6	420	<,001
shown_CPR_scenario	14,380	6	420	<,001

Probability distribution: Normal
Link function: Identity[a]
a. Target: number_additional_errors_identified

Fixed Effects

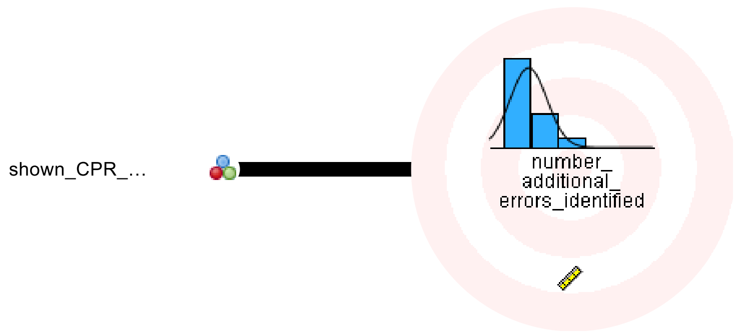

Fixed Coefficients[a]

Model Term	Coefficient	Std. Error	t	Sig.	95% Confidence Interval	
					Lower	Upper
Intercept	,918	,0799	11,495	<,001	,761	1,075
shown_CPR_scenario=1	-,836	,1129	-7,402	<,001	-1,058	-,614
shown_CPR_scenario=2	-,410	,1129	-3,629	<,001	-,632	-,188
shown_CPR_scenario=3	-,525	,1129	-4,645	<,001	-,747	-,303
shown_CPR_scenario=4	-,492	,1129	-4,354	<,001	-,714	-,270
shown_CPR_scenario=5	-,262	,1129	-2,322	,021	-,484	-,040
shown_CPR_scenario=6	-,852	,1129	-7,548	<,001	-1,074	-,630
shown_CPR_scenario=7	0[b]	.	.	.	.	.

Probability distribution: Normal

Link function: Identity[a]

a. Target: number_additional_errors_identified

b. This coefficient is set to zero because it is redundant.

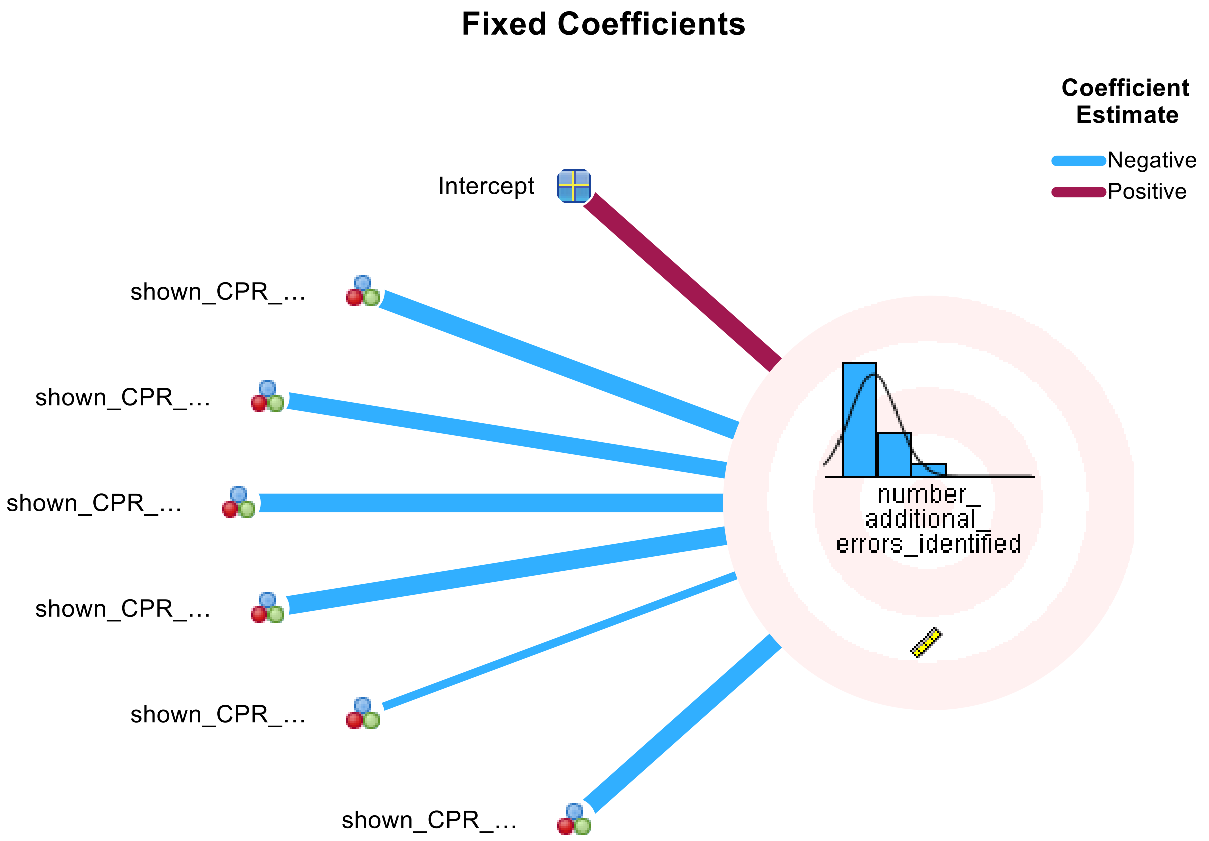

Fixed Coefficients
Coefficient Estimate
Negative
Positive
Intercept
shown_CPR_...
shown_CPR_...
shown_CPR_...
shown_CPR_...
shown_CPR_...
shown_CPR_...
number_ additional_ errors_identified

Covariances of Fixed Coefficients[a]

	Intercept	shown_CPR_scenario=1	shown_CPR_scenario=2	shown_CPR_scenario=3	shown_CPR_scenario=4
Intercept	,00638	-,00638	-,00638	-,00638	-,00638
shown_CPR_scenario=1	-,00638	,01276	,00638	,00638	,00638
shown_CPR_scenario=2	-,00638	,00638	,01276	,00638	,00638
shown_CPR_scenario=3	-,00638	,00638	,00638	,01276	,00638
shown_CPR_scenario=4	-,00638	,00638	,00638	,00638	,01276
shown_CPR_scenario=5	-,00638	,00638	,00638	,00638	,00638
shown_CPR_scenario=6	-,00638	,00638	,00638	,00638	,00638
shown_CPR_scenario=7	0[b]	0[b]	0[b]	0[b]	0[b]

Covariances of Fixed Coefficients[a]

	shown_CPR_scenario=5	shown_CPR_scenario=6	shown_CPR_scenario=7
Intercept	-,00638	-,00638	0[b]
shown_CPR_scenario=1	,00638	,00638	0[b]
shown_CPR_scenario=2	,00638	,00638	0[b]
shown_CPR_scenario=3	,00638	,00638	0[b]
shown_CPR_scenario=4	,00638	,00638	0[b]
shown_CPR_scenario=5	,01276	,00638	0[b]
shown_CPR_scenario=6	,00638	,01276	0[b]
shown_CPR_scenario=7	0[b]	0[b]	0[b]

Probability distribution: Normal

Link function: Identity[a]

a. Target: number_additional_errors_identified

b. One or both coefficients are redundant.

Correlations of Fixed Coefficients[a]

	Intercept	shown_CPR_scenario=1	shown_CPR_scenario=2	shown_CPR_scenario=3	shown_CPR_scenario=4
Intercept	1,000	-,707	-,707	-,707	-,707
shown_CPR_scenario=1	-,707	1,000	,500	,500	,500
shown_CPR_scenario=2	-,707	,500	1,000	,500	,500
shown_CPR_scenario=3	-,707	,500	,500	1,000	,500
shown_CPR_scenario=4	-,707	,500	,500	,500	1,000
shown_CPR_scenario=5	-,707	,500	,500	,500	,500
shown_CPR_scenario=6	-,707	,500	,500	,500	,500
shown_CPR_scenario=7	.[b]	.[b]	.[b]	.[b]	.[b]

Correlations of Fixed Coefficients[a]

	shown_CPR_scenario=5	shown_CPR_scenario=6	shown_CPR_scenario=7
Intercept	-,707	-,707	.[b]
shown_CPR_scenario=1	,500	,500	.[b]
shown_CPR_scenario=2	,500	,500	.[b]
shown_CPR_scenario=3	,500	,500	.[b]
shown_CPR_scenario=4	,500	,500	.[b]
shown_CPR_scenario=5	1,000	,500	.[b]
shown_CPR_scenario=6	,500	1,000	.[b]
shown_CPR_scenario=7	.[b]	.[b]	.[b]

Probability distribution: Normal

Link function: Identity[a]

a. Target: number_additional_errors_identified

b. One or both coefficients are redundant.

Covariance Parameters

Covariance Parameters Summary

Covariance Parameters	Residual Effect	1
	Random Effects	0
Design Matrix Columns	Fixed Effects	8
	Random Effects	0[a]
Common Subjects		1

Common subjects are based on the subject specifications for the residual and random effects and are used to chunk the data for better performance.

a. This is the number of columns per common subject.

Residual Effect

Residual Effect	Estimate	Std. Error	Z	Sig.	95% Confidence Interval Lower	95% Confidence Interval Upper
Variance	,389	,027	14,491	<,001	,340	,445

Covariance Structure: Scaled Identity

Subject Specification: (None)

Estimated Marginal Means for Top Significant Fixed Effects

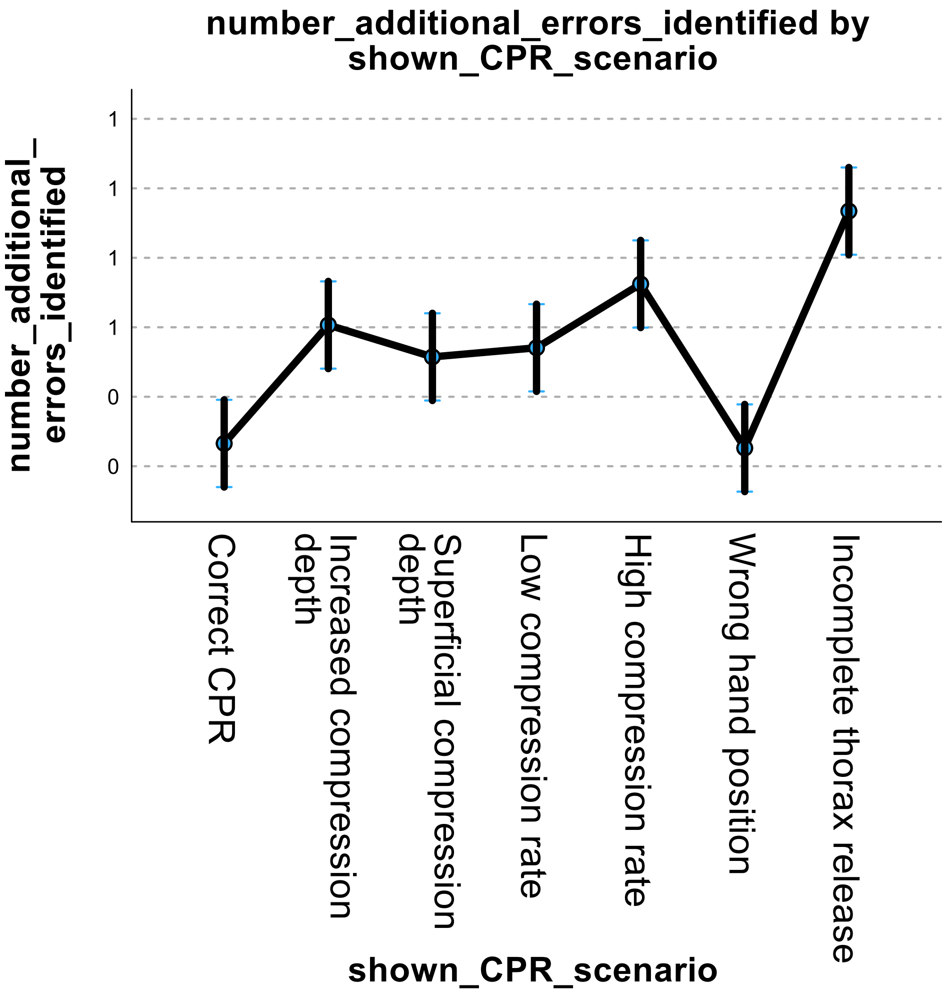

Supplement: Supplementary file 4 — Supplementary Material 4 [file 41598_2025_12306_MOESM4_ESM.pdf]
