# Supplementary material for: Influence of professional background on assessment of simulated cardiopulmonary resuscitation videos in an observational study
Source: Sci Rep. 2025 Jul 29;15:27648. doi: 10.1038/s41598-025-12306-x (PMC12307580; doi:10.1038/s41598-025-12306-x)
Supplement: Supplementary file 5 — Supplementary Material 5 [file 41598_2025_12306_MOESM5_ESM.pdf]

Supplementary Table S4

This document presents the original SPSS output for a series of linear mixed-effects regression models (GENLINMIXED) investigating factors influencing participants' ability to correctly classify CPR-related and Ventilation-related performance errors. The models assess whether demographic and professional characteristics (profession, age, gender, professional experience, number of passed CPR courses) are associated with classification accuracy. Since age is highly correlated with professional experience, we constructed two versions of the models for CPR and ventilation quality: one including age as a predictor and one excluding it. The SPSS syntax used for model estimation is presented first, followed by the corresponding output tables. All abbreviations and variable codings are explained in the scenario legend below. Reported results include fixed effects, confidence intervals, and model fit statistics.

I. Linear Mixed-Effects Model for CPR performance Classification

a. Predictors included in the model: profession, age, gender, professional experience number of CPR courses
   Predictors excluded: none

Scenario Legend

Shown CPR scenario 1	Correct CPR
Shown CPR scenario 2	Increased compression depth
Shown CPR scenario 3	Superficial compression depth
Shown CPR scenario 4	Low compression rate
Shown CPR scenario 5	High compression rate
Shown CPR scenario 6	Wrong hand position
Shown CPR scenario 7	Incomplete thorax release
Shown CPR scenario 8	Insufficient ventilation
Shown CPR scenario 9	Sufficient ventilation
Gender 1	Male
Gender 2	Female
Profession 1	Emergency medical service
Profession 2	Emergency physician

Syntax:

```
*Generalized Linear Mixed Models.
GENLINMIXED
  /DATA_STRUCTURE SUBJECTS=ID
  /FIELDS TARGET=correct_classification_CPR_only TRIALS=NONE OFFSET=NONE
  /TARGET_OPTIONS DISTRIBUTION=NORMAL LINK=IDENTITY
  /FIXED  EFFECTS=profession age gender professional_experience number_of_CPR_courses USE_INTERCEPT=TRUE
  /BUILD_OPTIONS TARGET_CATEGORY_ORDER=ASCENDING INPUTS_CATEGORY_ORDER=ASCENDING
HCONVERGE=0.00000001(RELATIVE) MAX_ITERATIONS=100 CONFIDENCE_LEVEL=95 DF_METHOD=RESIDUAL COVB=MODEL
SCORING=0 SINGULAR=0.000000000001
  /EMMEANS_OPTIONS SCALE=ORIGINAL PADJUST=LSD.
```

Generalized Linear Mixed Models

Warnings

Data Structure: One or more subject fields were specified but not actually used in the analysis.

Case Processing Summary

	N	Percent
Included	61	100,0%
Excluded	0	0,0%
Total	61	100,0%

Model Summary

Target		correct_classification_CPR_only
Probability Distribution		Normal
Link Function		Identity
Information Criterion	Akaike Corrected	201,605
	Bayesian	203,537

Information criteria are based on the -2 log likelihood (199,529) and are used to compare models. Models with smaller information criterion values fit better.

Coefficients of Determination

Pseudo-R Square Measures	Marginal	,032
	Conditional	,032

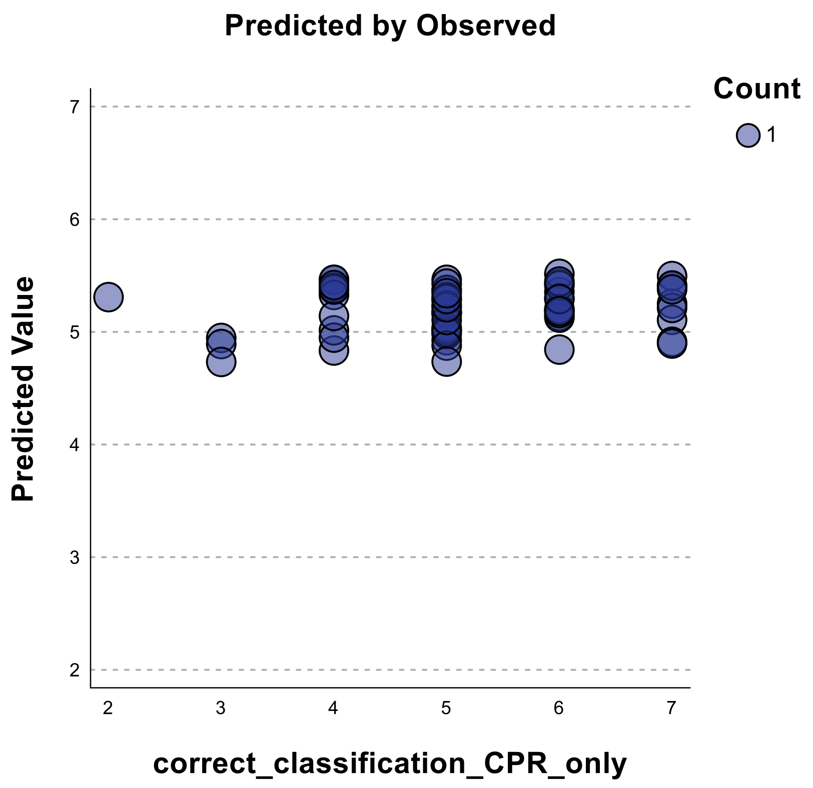

Fixed Effects[a]

Source	F	df1	df2	Sig.
Corrected Model	,367	5	55	,869
profession	,042	1	55	,838
age	,310	1	55	,580
gender	1,337	1	55	,253
professional_experience	,006	1	55	,938
number_of_CPR_cours es	,160	1	55	,691

Probability distribution: Normal

Link function: Identity[a]

a. Target: correct_classification_CPR_only

Fixed Effects

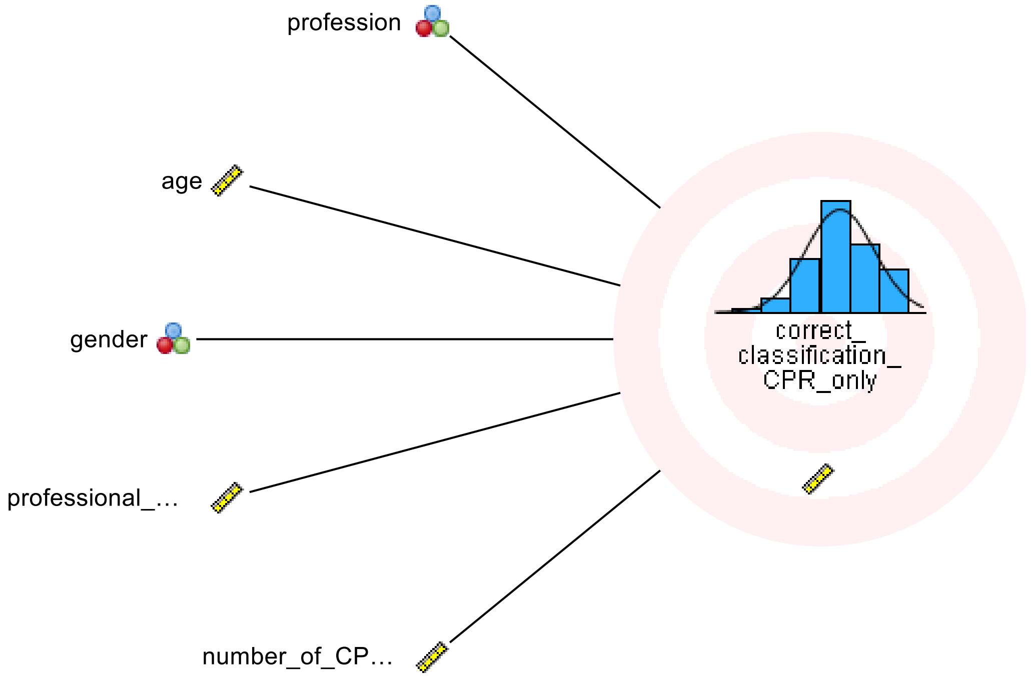

Fixed Coefficients[a]

Model Term	Coefficient	Std. Error	t	Sig.	95% Confidence Interval	
					Lower	Upper
Intercept	5,470	1,0529	5,195	<,001	3,360	7,580
profession=1	-,097	,4733	-,205	,838	-1,046	,852
profession=2	0[b]	.	.	.	.	.
age	-,018	,0323	-,557	,580	-,083	,047
gender=1	,448	,3874	1,156	,253	-,328	1,224
gender=2	0[b]	.	.	.	.	.
professional_experience	-,016	,2045	-,078	,938	-,426	,394
number_of_CPR_courses	,052	,1311	,400	,691	-,210	,315

Probability distribution: Normal
Link function: Identity[a]

a. Target: correct_classification_CPR_only

b. This coefficient is set to zero because it is redundant.

Fixed Coefficients

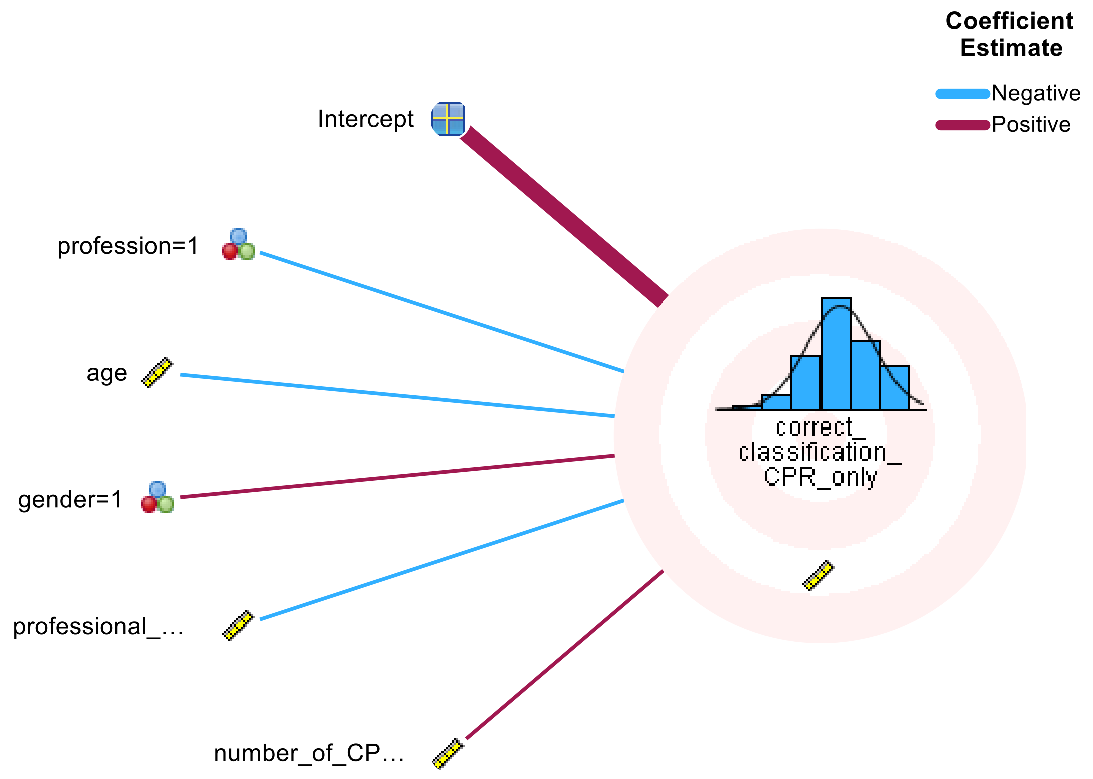

Covariances of Fixed Coefficients[a]

	Intercept	profession=1	profession=2	age	gender=1	gender=2
Intercept	1,10849	-,35214	0[b]	-,02764	-,03451	0[b]
profession=1	-,35214	,22404	0[b]	,00763	-,04018	0[b]
profession=2	0[b]	0[b]	0[b]	0[b]	0[b]	0[b]
age	-,02764	,00763	0[b]	,00105	-,00048	0[b]
gender=1	-,03451	-,04018	0[b]	-,00048	,15005	0[b]
gender=2	0[b]	0[b]	0[b]	0[b]	0[b]	0[b]
professional_experience	,06233	-,02106	0[b]	-,00441	-,01691	0[b]
number_of_CPR_courses	-,05739	,03493	0[b]	,00036	,00304	0[b]

Covariances of Fixed Coefficients[a]

	professional_experience	number_of_CPR_courses
Intercept	,06233	-,05739
profession=1	-,02106	,03493
profession=2	0[b]	0[b]
age	-,00441	,00036
gender=1	-,01691	,00304
gender=2	0[b]	0[b]
professional_experience	,04182	-,00274
number_of_CPR_courses	-,00274	,01719

Probability distribution: Normal
Link function: Identity[a]

a. Target: correct_classification_CPR_only

b. One or both coefficients are redundant.

Correlations of Fixed Coefficients[a]

	Intercept	profession=1	profession=2	age	gender=1	gender=2
Intercept	1,000	-,707	.[b]	-,812	-,085	.[b]
profession=1	-,707	1,000	.[b]	,498	-,219	.[b]
profession=2	.[b]	.[b]	.[b]	.[b]	.[b]	.[b]
age	-,812	,498	.[b]	1,000	-,039	.[b]
gender=1	-,085	-,219	.[b]	-,039	1,000	.[b]
gender=2	.[b]	.[b]	.[b]	.[b]	.[b]	.[b]
professional_experience	,289	-,218	.[b]	-,667	-,213	.[b]
number_of_CPR_courses	-,416	,563	.[b]	,085	,060	.[b]

Correlations of Fixed Coefficients[a]

	professional_experience	number_of_CPR_courses
Intercept	,289	-,416
profession=1	-,218	,563
profession=2	.[b]	.[b]
age	-,667	,085
gender=1	-,213	,060
gender=2	.[b]	.[b]
professional_experience	1,000	-,102
number_of_CPR_courses	-,102	1,000

Probability distribution: Normal
Link function: Identity[a]

a. Target: correct_classification_CPR_only

b. One or both coefficients are redundant.

Covariance Parameters

Covariance Parameters Summary

Covariance Parameters	Residual Effect	1
	Random Effects	0
Design Matrix Columns	Fixed Effects	8
	Random Effects	0[a]
Common Subjects		1

Common subjects are based on the subject specifications for the residual and random effects and are used to chunk the data for better performance.

a. This is the number of columns per common subject.

Residual Effect

Residual Effect	Estimate	Std. Error	Z	Sig.	95% Confidence Interval	
					Lower	Upper
Variance	1,401	,267	5,244	<,001	,964	2,036

Covariance Structure: Scaled Identity

Subject Specification: (None)

I. Linear Mixed-Effects Model for CPR performance Classification

b. Predictors included in the model: profession, gender, professional experience number of CPR courses
Predictor excluded: age

Scenario Legend

Shown CPR scenario 1	Correct CPR
Shown CPR scenario 2	Increased compression depth
Shown CPR scenario 3	Superficial compression depth
Shown CPR scenario 4	Low compression rate
Shown CPR scenario 5	High compression rate
Shown CPR scenario 6	Wrong hand position
Shown CPR scenario 7	Incomplete thorax release
Shown CPR scenario 8	Insufficient ventilation
Shown CPR scenario 9	Sufficient ventilation
Gender 1	Male
Gender 2	Female
Profession 1	Emergency medical service
Profession 2	Emergency physician

Syntax:

```
*Generalized Linear Mixed Models.
GENLINMIXED
  /DATA_STRUCTURE SUBJECTS=ID
  /FIELDS TARGET=correct_classification_CPR_only TRIALS=NONE OFFSET=NONE
  /TARGET_OPTIONS DISTRIBUTION=NORMAL LINK=IDENTITY
  /FIXED  EFFECTS=profession gender professional_experience number_of_CPR_courses USE_INTERCEPT=TRUE
  /BUILD_OPTIONS TARGET_CATEGORY_ORDER=ASCENDING INPUTS_CATEGORY_ORDER=ASCENDING
HCONVERGE=0.00000001(RELATIVE) MAX_ITERATIONS=100 CONFIDENCE_LEVEL=95 DF_METHOD=RESIDUAL COVB=MODEL
SCORING=0 SINGULAR=0.000000000001
  /EMMEANS_OPTIONS SCALE=ORIGINAL PADJUST=LSD.
```

Generalized Linear Mixed Models

Warnings

Data Structure: One or more subject fields were specified but not actually used in the analysis.

Case Processing Summary

	N	Percent
Included	61	100,0%
Excluded	0	0,0%
Total	61	100,0%

Model Summary

Target		correct_classification_CPR_only
Probability Distribution		Normal
Link Function		Identity
Information Criterion	Akaike Corrected	196,884
	Bayesian	198,836

Information criteria are based on the -2 log likelihood (194,810) and are used to compare models. Models with smaller information criterion values fit better.

Coefficients of Determination

Pseudo-R Square Measures	Marginal	,027
	Conditional	,027

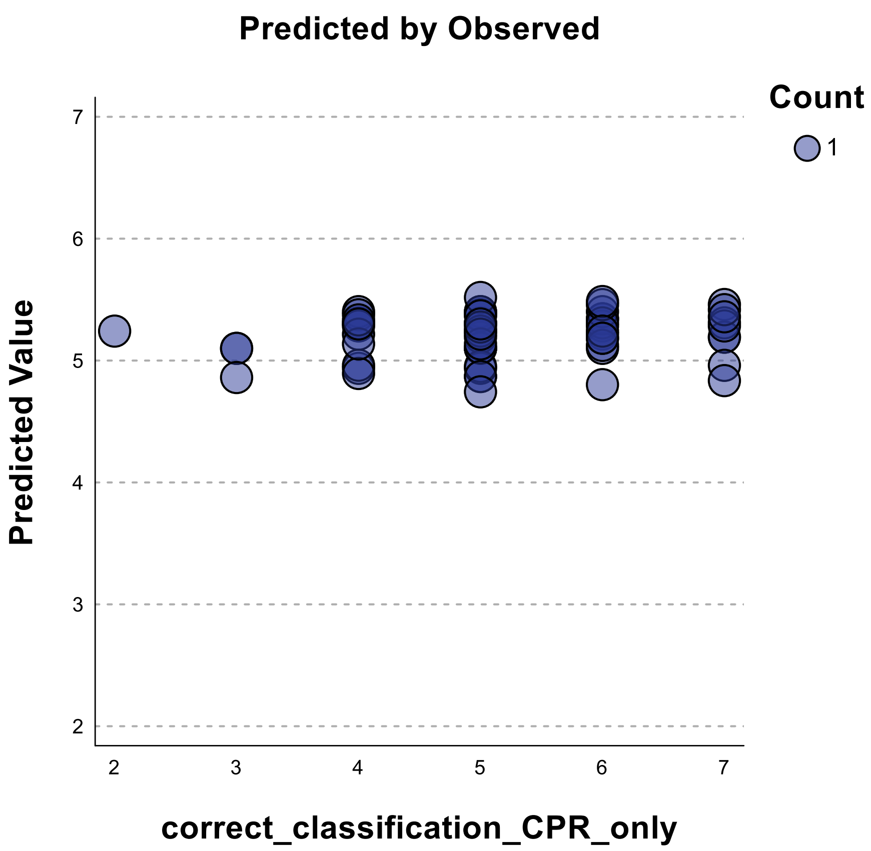

Fixed Effects[a]

Source	F	df1	df2	Sig.
Corrected Model	,386	4	56	,818
profession	,007	1	56	,933
gender	1,306	1	56	,258
professional_experience	,368	1	56	,546
number_of_CPR_courses	,204	1	56	,653

Probability distribution: Normal
Link function: Identity[a]
a. Target: correct_classification_CPR_only

Fixed Effects

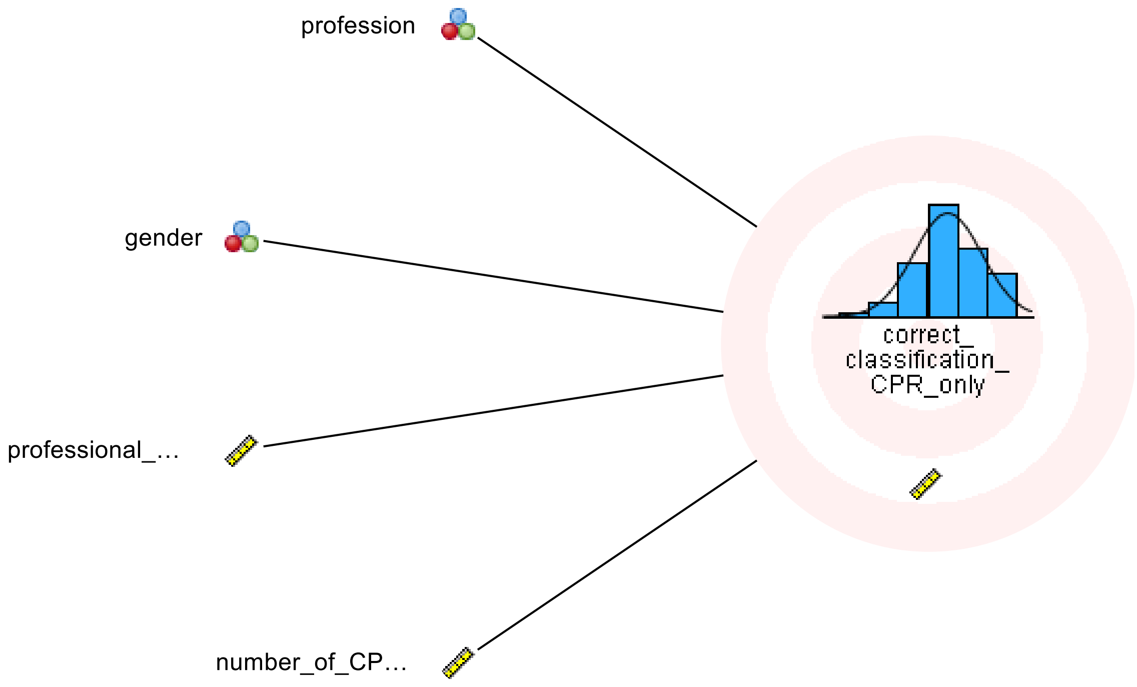

Fixed Coefficients[a]

Model Term	Coefficient	Std. Error	t	Sig.	95% Confidence Interval	
					Lower	Upper
Intercept	4,994	,6111	8,173	<,001	3,770	6,218
profession=1	,034	,4078	,084	,933	-,783	,851
profession=2	0[b]	.	.	.	.	.
gender=1	,440	,3847	1,143	,258	-,331	1,210
gender=2	0[b]	.	.	.	.	.
professional_experience	-,092	,1515	-,607	,546	-,395	,212
number_of_CPR_courses	,059	,1298	,452	,653	-,201	,319

Probability distribution: Normal
Link function: Identity[a]

a. Target: correct_classification_CPR_only

b. This coefficient is set to zero because it is redundant.

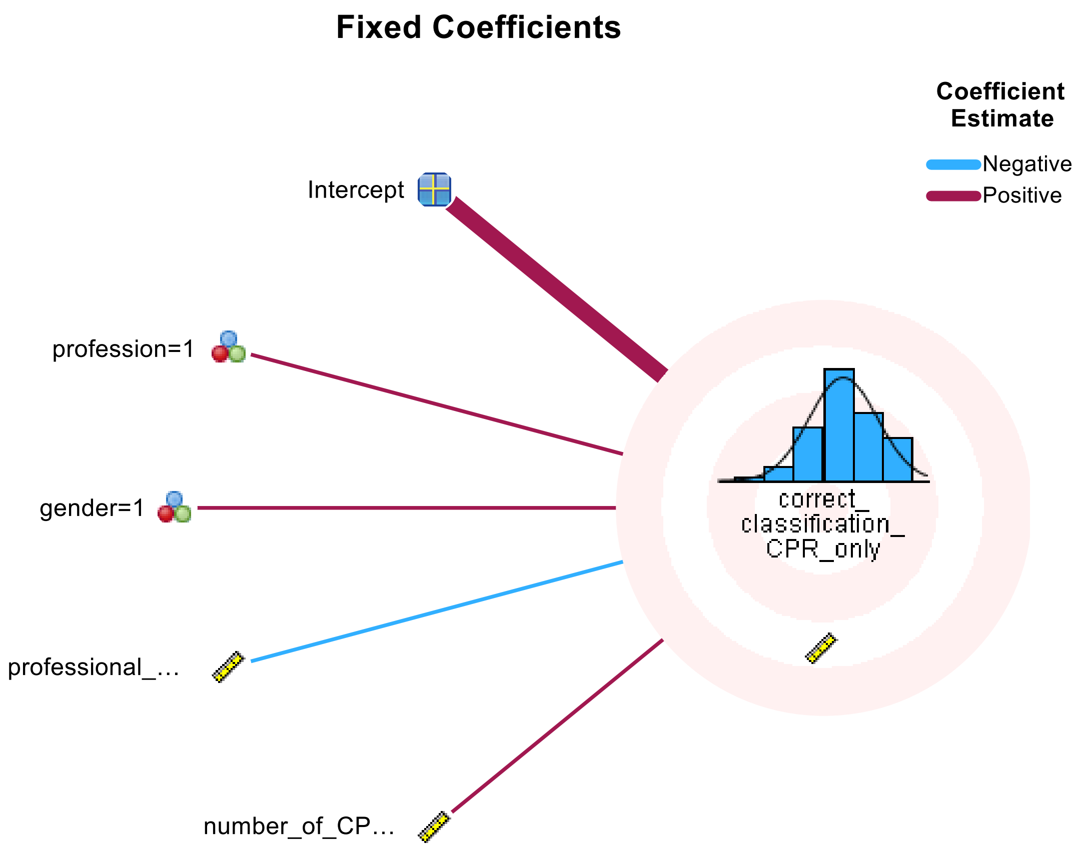

Fixed Coefficients
Coefficient Estimate
Negative
Positive
Intercept
profession=1
gender=1
professional_...
number_of_CP...
correct_ classification_ CPR_only

Covariances of Fixed Coefficients[a]

	Intercept	profession=1	profession=2	gender=1	gender=2
Intercept	,37339	-,14863	0[b]	-,04674	0[b]
profession=1	-,14863	,16630	0[b]	-,03619	0[b]
profession=2	0[b]	0[b]	0[b]	0[b]	0[b]
gender=1	-,04674	-,03619	0[b]	,14798	0[b]
gender=2	0[b]	0[b]	0[b]	0[b]	0[b]
professional_experience	-,05351	,01097	0[b]	-,01872	0[b]
number_of_CPR_courses	-,04728	,03190	0[b]	,00317	0[b]

Covariances of Fixed Coefficients[a]

	professional_experience	number_of_CPR_courses
Intercept	-,05351	-,04728
profession=1	,01097	,03190
profession=2	0[b]	0[b]
gender=1	-,01872	,00317
gender=2	0[b]	0[b]
professional_experience	,02295	-,00120
number_of_CPR_courses	-,00120	,01686

Probability distribution: Normal

Link function: Identity[a]

a. Target: correct_classification_CPR_only

b. One or both coefficients are redundant.

Correlations of Fixed Coefficients[a]

	Intercept	profession=1	profession=2	gender=1	gender=2
Intercept	1,000	-,596	.[b]	-,199	.[b]
profession=1	-,596	1,000	.[b]	-,231	.[b]
profession=2	.[b]	.[b]	.[b]	.[b]	.[b]
gender=1	-,199	-,231	.[b]	1,000	.[b]
gender=2	.[b]	.[b]	.[b]	.[b]	.[b]
professional_experience	-,578	,178	.[b]	-,321	.[b]
number_of_CPR_courses	-,596	,603	.[b]	,063	.[b]

Correlations of Fixed Coefficients[a]

	professional_experience	number_of_CPR_courses
Intercept	-,578	-,596
profession=1	,178	,603
profession=2	.[b]	.[b]
gender=1	-,321	,063
gender=2	.[b]	.[b]
professional_experience	1,000	-,061
number_of_CPR_courses	-,061	1,000

Probability distribution: Normal

Link function: Identity[a]

a. Target: correct_classification_CPR_only

b. One or both coefficients are redundant.

Covariance Parameters

Covariance Parameters Summary

Covariance Parameters	Residual Effect	1
	Random Effects	0
Design Matrix Columns	Fixed Effects	7
	Random Effects	0[a]
Common Subjects		1

Common subjects are based on the subject specifications for the residual and random effects and are used to chunk the data for better performance.

a. This is the number of columns per common subject.

Residual Effect

Residual Effect	Estimate	Std. Error	Z	Sig.	95% Confidence Interval	
					Lower	Upper
Variance	1,384	,262	5,292	<,001	,956	2,004

Covariance Structure: Scaled Identity

Subject Specification: (None)

II. Linear Mixed-Effects Model for <u>Ventilation</u> performance Classification

a. <u>Predictors included in the model</u>: profession, age, gender, professional experience number of CPR courses
b. <u>Predictors excluded</u>: none

Scenario Legend

Shown CPR scenario 1	Correct CPR
Shown CPR scenario 2	Increased compression depth
Shown CPR scenario 3	Superficial compression depth
Shown CPR scenario 4	Low compression rate
Shown CPR scenario 5	High compression rate
Shown CPR scenario 6	Wrong hand position
Shown CPR scenario 7	Incomplete thorax release
Shown CPR scenario 8	Insufficient ventilation
Shown CPR scenario 9	Sufficient ventilation
Gender 1	Male
Gender 2	Female
Profession 1	Emergency medical service
Profession 2	Emergency physician

Syntax:

```
*Generalized Linear Mixed Models.
GENLINMIXED
  /DATA_STRUCTURE SUBJECTS=ID
  /FIELDS TARGET=correct_classification_ventilation_only TRIALS=NONE OFFSET=NONE
  /TARGET_OPTIONS DISTRIBUTION=NORMAL LINK=IDENTITY
  /FIXED  EFFECTS=profession age gender professional_experience number_of_CPR_courses USE_INTERCEPT=TRUE
  /BUILD_OPTIONS TARGET_CATEGORY_ORDER=ASCENDING INPUTS_CATEGORY_ORDER=ASCENDING
HCONVERGE=0.00000001(RELATIVE) MAX_ITERATIONS=100 CONFIDENCE_LEVEL=95 DF_METHOD=RESIDUAL COVB=MODEL
SCORING=0 SINGULAR=0.000000000001
  /EMMEANS_OPTIONS SCALE=ORIGINAL PADJUST=LSD.
```

Generalized Linear Mixed Models

Warnings

Data Structure: One or more subject fields were specified but not actually used in the analysis.

Case Processing Summary

	N	Percent
Included	61	100,0%
Excluded	0	0,0%
Total	61	100,0%

Model Summary

Target		correct_classification_ventilation_only
Probability Distribution		Normal
Link Function		Identity
Information Criterion	Akaike Corrected	58,997
	Bayesian	60,929

Information criteria are based on the -2 log likelihood (56,922) and are used to compare models. Models with smaller information criterion values fit better.

Coefficients of Determination

Pseudo-R Square Measures	Marginal	,125
	Conditional	,125

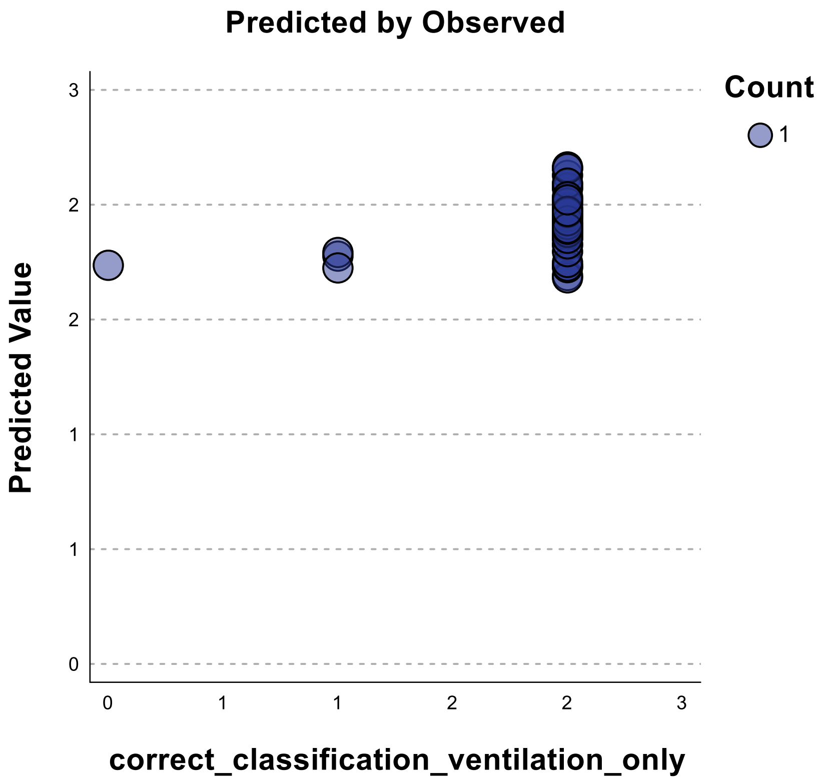

Fixed Effects[a]

Source	F	df1	df2	Sig.
Corrected Model	1,574	5	55	,183
profession	,430	1	55	,515
age	,150	1	55	,700
gender	,026	1	55	,873
professional_experience	,280	1	55	,599
number_of_CPR_courses	2,516	1	55	,118

Probability distribution: Normal

Link function: Identity[a]

a. Target: correct_classification_ventilation_only

Fixed Effects

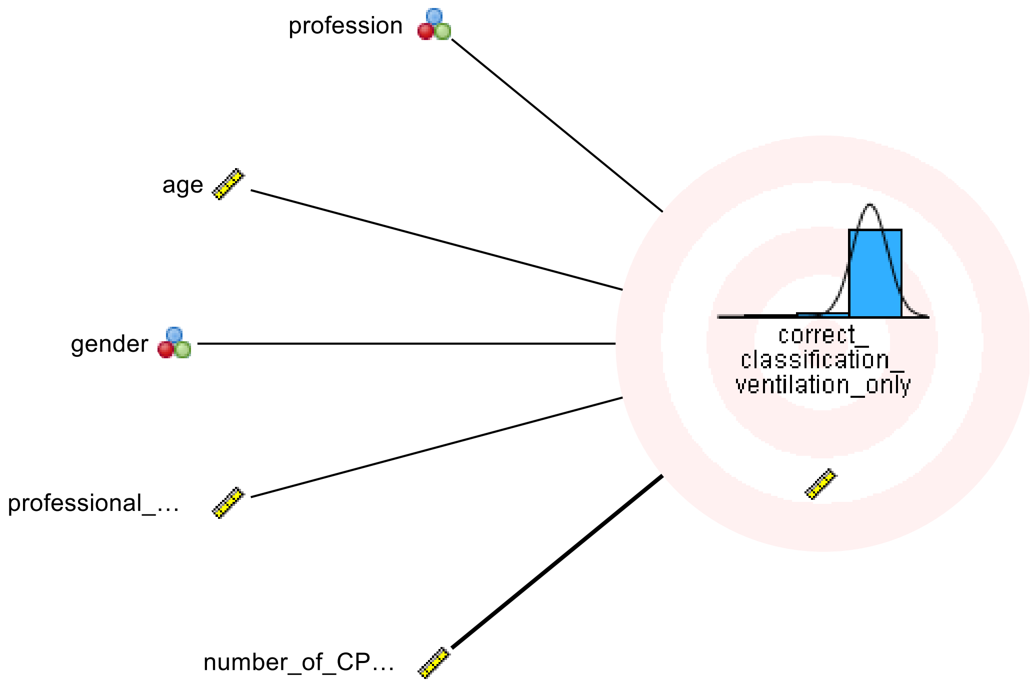

Fixed Coefficients[a]

Model Term	Coefficient	Std. Error	t	Sig.	95% Confidence Interval	
					Lower	Upper
Intercept	2,066	,2880	7,175	<,001	1,489	2,643
profession=1	-,085	,1295	-,655	,515	-,344	,175
profession=2	0[b]	.	.	.	.	.
age	-,003	,0088	-,387	,700	-,021	,014
gender=1	-,017	,1059	-,160	,873	-,229	,195
gender=2	0[b]	.	.	.	.	.
professional_experience	-,030	,0559	-,529	,599	-,142	,083
number_of_CPR_courses	,057	,0359	1,586	,118	-,015	,129

Probability distribution: Normal
Link function: Identity[a]

a. Target: correct_classification_ventilation_only

b. This coefficient is set to zero because it is redundant.

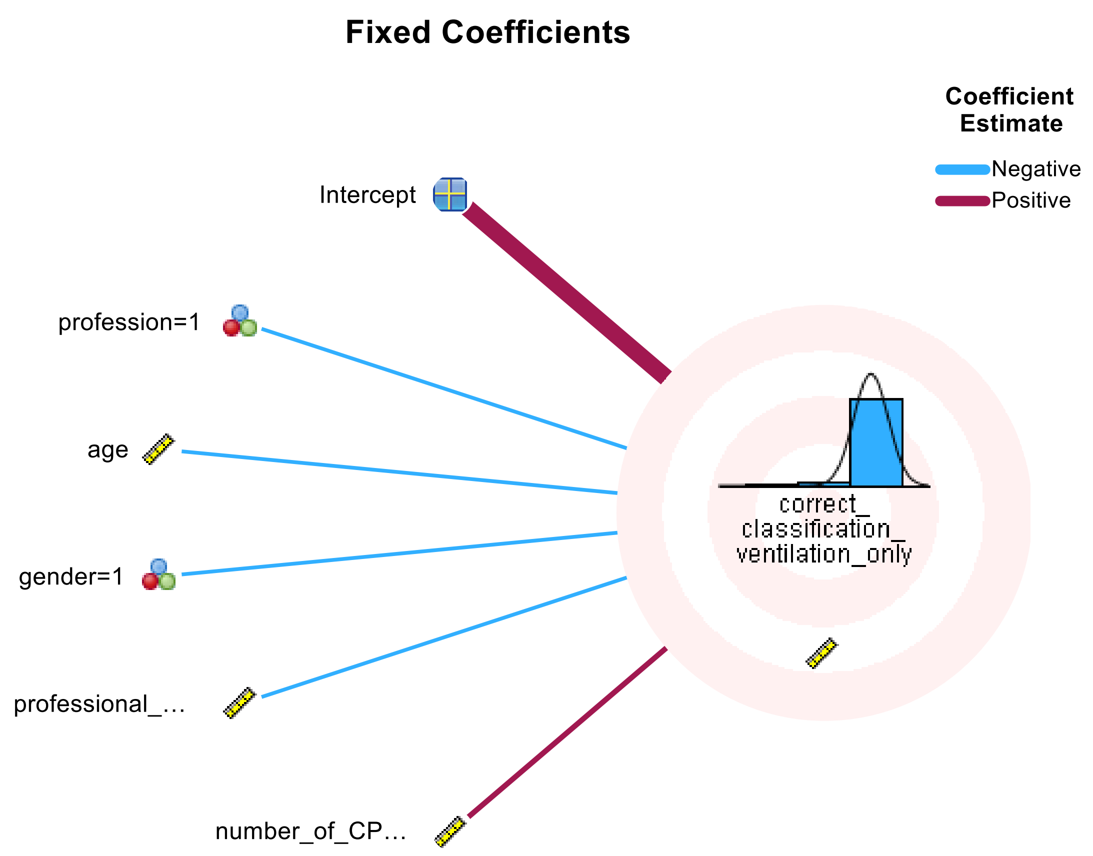
Fixed Coefficients
Coefficient Estimate
Negative
Positive
Intercept
profession=1
age
gender=1
professional_...
number_of_CP...
correct_
classification_
ventilation_only

Covariances of Fixed Coefficients[a]

	Intercept	profession=1	profession=2	age	gender=1
Intercept	,08292	-,02634	0[b]	-,00207	-,00258
profession=1	-,02634	,01676	0[b]	,00057	-,00301
profession=2	0[b]	0[b]	0[b]	0[b]	0[b]
age	-,00207	,00057	0[b]	7,82456E-5	-3,62802E-5
gender=1	-,00258	-,00301	0[b]	-3,62802E-5	,01122
gender=2	0[b]	0[b]	0[b]	0[b]	0[b]
professional_experience	,00466	-,00158	0[b]	-,00033	-,00126
number_of_CPR_courses	-,00429	,00261	0[b]	2,69270E-5	,00023

Covariances of Fixed Coefficients[a]

	gender=2	professional_experience	number_of_CPR_courses
Intercept	0[b]	,00466	-,00429
profession=1	0[b]	-,00158	,00261
profession=2	0[b]	0[b]	0[b]
age	0[b]	-,00033	2,69270E-5
gender=1	0[b]	-,00126	,00023
gender=2	0[b]	0[b]	0[b]
professional_experience	0[b]	,00313	-,00020
number_of_CPR_courses	0[b]	-,00020	,00129

Probability distribution: Normal

Link function: Identity[a]

a. Target: correct_classification_ventilation_only

b. One or both coefficients are redundant.

Correlations of Fixed Coefficients[a]

	Intercept	profession=1	profession=2	age	gender=1	gender=2
Intercept	1,000	-,707	.[b]	-,812	-,085	.[b]
profession=1	-,707	1,000	.[b]	,498	-,219	.[b]
profession=2	.[b]	.[b]	.[b]	.[b]	.[b]	.[b]
age	-,812	,498	.[b]	1,000	-,039	.[b]
gender=1	-,085	-,219	.[b]	-,039	1,000	.[b]
gender=2	.[b]	.[b]	.[b]	.[b]	.[b]	.[b]
professional_experience	,289	-,218	.[b]	-,667	-,213	.[b]
number_of_CPR_courses	-,416	,563	.[b]	,085	,060	.[b]

Correlations of Fixed Coefficients[a]

	professional_experience	number_of_CPR_courses
Intercept	,289	-,416
profession=1	-,218	,563
profession=2	.[b]	.[b]
age	-,667	,085
gender=1	-,213	,060
gender=2	.[b]	.[b]
professional_experience	1,000	-,102
number_of_CPR_courses	-,102	1,000

Probability distribution: Normal

Link function: Identity[a]

a. Target: correct_classification_ventilation_only

b. One or both coefficients are redundant.

Covariance Parameters

Covariance Parameters Summary

Covariance Parameters	Residual Effect	1
	Random Effects	0
Design Matrix Columns	Fixed Effects	8
	Random Effects	0[a]
Common Subjects		1

Common subjects are based on the subject specifications for the residual and random effects and are used to chunk the data for better performance.

a. This is the number of columns per common subject.

Residual Effect

Residual Effect	Estimate	Std. Error	Z	Sig.	95% Confidence Interval	
					Lower	Upper
Variance	,105	,020	5,244	<,001	,072	,152

II. Linear Mixed-Effects Model for Ventilation performance classification

b. Predictors included in the model: profession, age, gender, professional experience number of CPR courses
Predictor excluded: age

Scenario Legend

Shown CPR scenario 1	Correct CPR
Shown CPR scenario 2	Increased compression depth
Shown CPR scenario 3	Superficial compression depth
Shown CPR scenario 4	Low compression rate
Shown CPR scenario 5	High compression rate
Shown CPR scenario 6	Wrong hand position
Shown CPR scenario 7	Incomplete thorax release
Shown CPR scenario 8	Insufficient ventilation
Shown CPR scenario 9	Sufficient ventilation
Gender 1	Male
Gender 2	Female
Profession 1	Emergency medical service
Profession 2	Emergency physician

Syntax:

```
*Generalized Linear Mixed Models.
GENLINMIXED
  /DATA_STRUCTURE SUBJECTS=ID
  /FIELDS TARGET=correct_classification_ventilation_only TRIALS=NONE OFFSET=NONE
  /TARGET_OPTIONS DISTRIBUTION=NORMAL LINK=IDENTITY
  /FIXED  EFFECTS=profession gender professional_experience number_of_CPR_courses USE_INTERCEPT=TRUE
  /BUILD_OPTIONS TARGET_CATEGORY_ORDER=ASCENDING INPUTS_CATEGORY_ORDER=ASCENDING
HCONVERGE=0.00000001(RELATIVE) MAX_ITERATIONS=100 CONFIDENCE_LEVEL=95 DF_METHOD=RESIDUAL COVB=MODEL
SCORING=0 SINGULAR=0.000000000001
  /EMMEANS_OPTIONS SCALE=ORIGINAL PADJUST=LSD.
```

Generalized Linear Mixed Models

Warnings

Data Structure: One or more subject fields were specified but not actually used in the analysis.

Case Processing Summary

	N	Percent
Included	61	100,0%
Excluded	0	0,0%
Total	61	100,0%

Model Summary

Target		correct_classification_ventilation_only
Probability Distribution		Normal
Link Function		Identity
Information Criterion	Akaike Corrected	51,522
	Bayesian	53,473

Information criteria are based on the -2 log likelihood (49,448) and are used to compare models. Models with smaller information criterion values fit better.

Coefficients of Determination

Pseudo-R Square Measures	Marginal	,123
	Conditional	,123

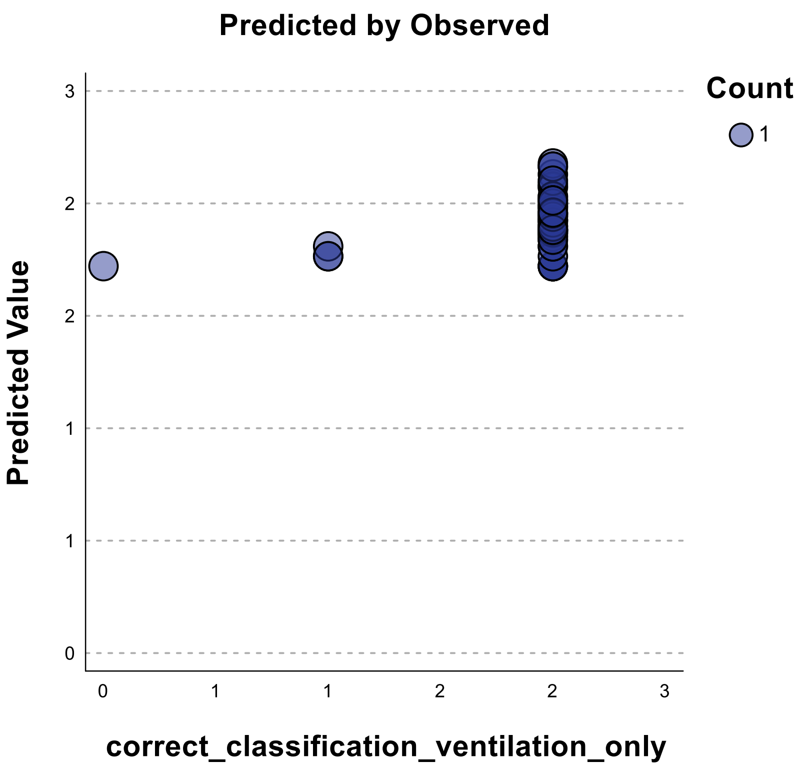

Fixed Effects[a]

Source	F	df1	df2	Sig.
Corrected Model	1,960	4	56	,113
profession	,289	1	56	,593
gender	,031	1	56	,860
professional_experience	1,132	1	56	,292
number_of_CPR_courses	2,681	1	56	,107

Probability distribution: Normal
Link function: Identity[a]
a. Target: correct_classification_ventilation_only

Fixed Effects

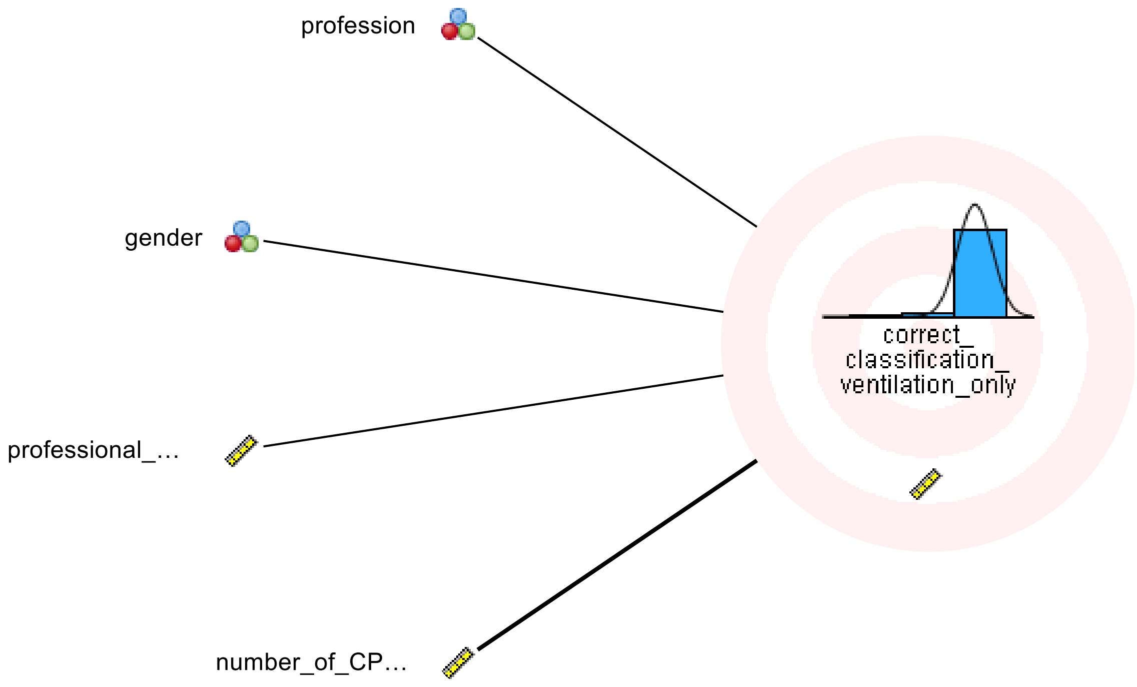

Fixed Coefficients[a]

Model Term	Coefficient	Std. Error	t	Sig.	95% Confidence Interval	
					Lower	Upper
Intercept	1,975	,1669	11,837	<,001	1,641	2,310
profession=1	-,060	,1114	-,537	,593	-,283	,163
profession=2	0[b]	.	.	.	.	.
gender=1	-,019	,1051	-,177	,860	-,229	,192
gender=2	0[b]	.	.	.	.	.
professional_experience	-,044	,0414	-1,064	,292	-,127	,039
number_of_CPR_courses	,058	,0355	1,637	,107	-,013	,129

Probability distribution: Normal

Link function: Identity[a]

a. Target: correct_classification_ventilation_only

b. This coefficient is set to zero because it is redundant.

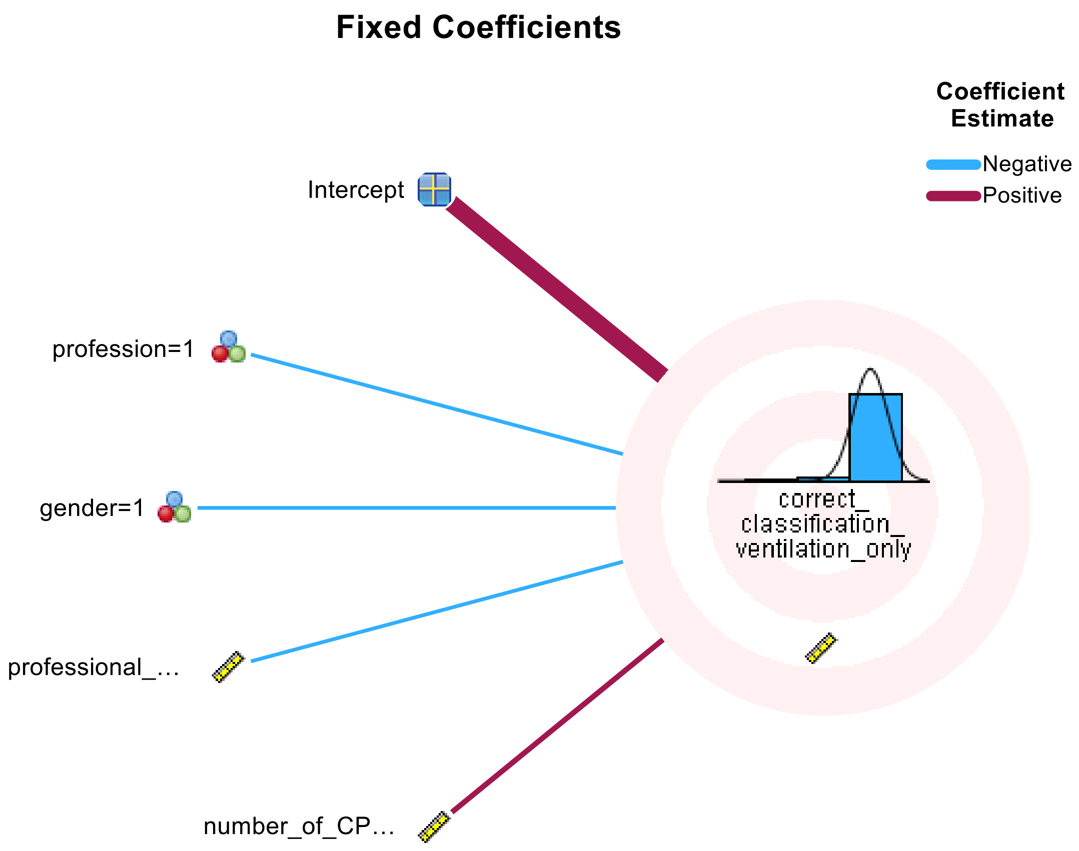

Fixed Coefficients
Coefficient Estimate
Negative
Positive
Intercept
profession=1
gender=1
professional_...
number_of_CP...
correct_ classification_ ventilation_only

Covariances of Fixed Coefficients[a]

	Intercept	profession=1	profession=2	gender=1	gender=2
Intercept	,02785	-,01109	0[b]	-,00349	0[b]
profession=1	-,01109	,01240	0[b]	-,00270	0[b]
profession=2	0[b]	0[b]	0[b]	0[b]	0[b]
gender=1	-,00349	-,00270	0[b]	,01104	0[b]
gender=2	0[b]	0[b]	0[b]	0[b]	0[b]
professional_experience	-,00399	,00082	0[b]	-,00140	0[b]
number_of_CPR_courses	-,00353	,00238	0[b]	,00024	0[b]

Covariances of Fixed Coefficients[a]

	professional_experience	number_of_CPR_courses
Intercept	-,00399	-,00353
profession=1	,00082	,00238
profession=2	0[b]	0[b]
gender=1	-,00140	,00024
gender=2	0[b]	0[b]
professional_experience	,00171	-8,98518E-5
number_of_CPR_courses	-8,98518E-5	,00126

Probability distribution: Normal
Link function: Identity[a]

a. Target: correct_classification_ventilation_only

b. One or both coefficients are redundant.

Correlations of Fixed Coefficients[a]

	Intercept	profession=1	profession=2	gender=1	gender=2
Intercept	1,000	-,596	.[b]	-,199	.[b]
profession=1	-,596	1,000	.[b]	-,231	.[b]
profession=2	.[b]	.[b]	.[b]	.[b]	.[b]
gender=1	-,199	-,231	.[b]	1,000	.[b]
gender=2	.[b]	.[b]	.[b]	.[b]	.[b]
professional_experience	-,578	,178	.[b]	-,321	.[b]
number_of_CPR_courses	-,596	,603	.[b]	,063	.[b]

Correlations of Fixed Coefficients[a]

	professional_experience	number_of_CPR_courses
Intercept	-,578	-,596
profession=1	,178	,603
profession=2	.[b]	.[b]
gender=1	-,321	,063
gender=2	.[b]	.[b]
professional_experience	1,000	-,061
number_of_CPR_courses	-,061	1,000

Probability distribution: Normal
Link function: Identity[a]

a. Target: correct_classification_ventilation_only

b. One or both coefficients are redundant.

Covariance Parameters

Covariance Parameters Summary

Covariance Parameters	Residual Effect	1
	Random Effects	0
Design Matrix Columns	Fixed Effects	7
	Random Effects	0[a]
Common Subjects		1

Common subjects are based on the subject specifications for the residual and random effects and are used to chunk the data for better performance.

a. This is the number of columns per common subject.

Residual Effect

Residual Effect	Estimate	Std. Error	Z	Sig.	95% Confidence Interval Lower	95% Confidence Interval Upper
Variance	,103	,020	5,292	<,001	,071	,150

Covariance Structure: Scaled Identity

Subject Specification: (None)
