# Supplementary material for: Influence of professional background on assessment of simulated cardiopulmonary resuscitation videos in an observational study
Source: Sci Rep. 2025 Jul 29;15:27648. doi: 10.1038/s41598-025-12306-x (PMC12307580; doi:10.1038/s41598-025-12306-x)
Supplement: Supplementary file 6 — Supplementary Material 6 [file 41598_2025_12306_MOESM6_ESM.pdf]

# Supplementary Table S5

This document includes the original SPSS output from a correlation analysis between age, number of passed CPR courses and professional experience, as well as a multicollinearity assessment of demographic and professional variables (profession, age, gender, professional experience, and number of completed CPR courses) on participants' ability to accurately identify CPR scenarios. The SPSS syntax used for model estimation is presented first, followed by the corresponding output tables. All abbreviations and variable codings are explained in the scenario legend below.

## a. **Correlation analysis of participant characteristics**

Scenario Legend

| | |
|---|---|
| Shown CPR scenario 1 | Correct CPR |
| Shown CPR scenario 2 | Increased compression depth |
| Shown CPR scenario 3 | Superficial compression depth |
| Shown CPR scenario 4 | Low compression rate |
| Shown CPR scenario 5 | High compression rate |
| Shown CPR scenario 6 | Wrong hand position |
| Shown CPR scenario 7 | Incomplete thorax release |
| Shown CPR scenario 8 | Insufficient ventilation |
| Shown CPR scenario 9 | Sufficient ventilation |
| Gender 1 | Male |
| Gender 2 | Female |
| Profession 1 | Emergency medical service |
| Profession 2 | Emergency physician |

Syntax:

```
STATS CORRELATIONS VARIABLES=age professional_experience number_of_CPR_courses
/OPTIONS CONFLEVEL=95 METHOD=FISHER
/MISSING EXCLUDE=YES PAIRWISE=YES.
```

Correlations

## Correlations

| Variable | Variable2 | Statistic: Correlation | Count | Lower C.I. | Upper C.I. |
|---|---|---|---|---|---|
| age | age | 1,000 | 61 | -- | -- |
| | professional_experience | ,670 | 61 | ,503 | ,789 |
| | number_of_CPR_courses | ,321 | 61 | ,075 | ,530 |
| number_of_CPR_courses | age | ,321 | 61 | ,075 | ,530 |
| | professional_experience | ,149 | 61 | -,106 | ,387 |
| | number_of_CPR_courses | 1,000 | 61 | -- | -- |
| professional_experience | age | ,670 | 61 | ,503 | ,789 |
| | professional_experience | 1,000 | 61 | -- | -- |
| | number_of_CPR_courses | ,149 | 61 | -,106 | ,387 |

## Correlations

| Variable | Variable2 | Statistic: Notes |
|---|---|---|
| age | age | |
| | professional_experience | |
| | number_of_CPR_courses | |
| number_of_CPR_courses | age | |
| | professional_experience | |
| | number_of_CPR_courses | |
| professional_experience | age | |
| | professional_experience | |
| | number_of_CPR_courses | |

Missing value handling: PAIRWISE, EXCLUDE. C.I. Level: 95.0

b. **Multicollinearity analysis**

```
Syntax:

REGRESSION
  /DESCRIPTIVES MEAN STDDEV CORR SIG N
  /MISSING LISTWISE
  /STATISTICS COEFF OUTS CI(95) BCOV R ANOVA COLLIN TOL CHANGE
  /CRITERIA=PIN(.05) POUT(.10)
  /NOORIGIN
  /DEPENDENT correct_classification_CPR_only
  /METHOD=ENTER age gender profession professional_experience number_of_CPR_courses.
```

Regression

### Descriptive Statistics

| | Mean | Std. Deviation | N |
|---|---|---|---|
| correct_classification_CPR_only | 5,20 | 1,152 | 61 |
| age | 34,23 | 7,727 | 61 |
| gender | 1,25 | ,434 | 61 |
| profession | 1,49 | ,504 | 61 |
| professional_experience | 2,80 | 1,077 | 61 |
| number_of_CPR_courses | 1,90 | 1,535 | 61 |

## Correlations

| | | correct_classification_CPR_only | age | gender | profession |
|---|---|---|---|---|---|
| Pearson Correlation | correct_classification_CPR_only | 1,000 | -,076 | -,132 | -,026 |
| | age | -,076 | 1,000 | -,057 | ,531 |
| | gender | -,132 | -,057 | 1,000 | ,276 |
| | profession | -,026 | ,531 | ,276 | 1,000 |
| | professional_experience | -,035 | ,670 | -,251 | ,181 |
| | number_of_CPR_courses | ,021 | ,321 | ,212 | ,645 |
| Sig. (1-tailed) | correct_classification_CPR_only | . | ,279 | ,156 | ,422 |
| | age | ,279 | . | ,332 | ,000 |
| | gender | ,156 | ,332 | . | ,016 |
| | profession | ,422 | ,000 | ,016 | . |
| | professional_experience | ,393 | ,000 | ,025 | ,081 |
| | number_of_CPR_courses | ,438 | ,006 | ,051 | ,000 |
| N | correct_classification_CPR_only | 61 | 61 | 61 | 61 |
| | age | 61 | 61 | 61 | 61 |
| | gender | 61 | 61 | 61 | 61 |
| | profession | 61 | 61 | 61 | 61 |
| | professional_experience | 61 | 61 | 61 | 61 |
| | number_of_CPR_courses | 61 | 61 | 61 | 61 |

## Correlations

| | | professional_experience | number_of_CPR_courses |
|---|---|---|---|
| Pearson Correlation | correct_classification_CPR_only | -,035 | ,021 |
| | age | ,670 | ,321 |
| | gender | -,251 | ,212 |
| | profession | ,181 | ,645 |
| | professional_experience | 1,000 | ,149 |
| | number_of_CPR_courses | ,149 | 1,000 |
| Sig. (1-tailed) | correct_classification_CPR_only | ,393 | ,438 |
| | age | ,000 | ,006 |
| | gender | ,025 | ,051 |
| | profession | ,081 | ,000 |
| | professional_experience | . | ,125 |
| | number_of_CPR_courses | ,125 | . |
| N | correct_classification_CPR_only | 61 | 61 |
| | age | 61 | 61 |
| | gender | 61 | 61 |
| | profession | 61 | 61 |
| | professional_experience | 61 | 61 |
| | number_of_CPR_courses | 61 | 61 |

## Variables Entered/Removed[a]

| Model | Variables Entered | Variables Removed | Method |
|---|---|---|---|
| 1 | number_of_CPR_courses, professional_experience, gender, profession, age[b] | . | Enter |

a. Dependent Variable: correct_classification_CPR_only

b. All requested variables entered.

## Model Summary

| | | | | | Change Statistics | | |
|---|---|---|---|---|---|---|---|
| Model | R | R Square | Adjusted R Square | Std. Error of the Estimate | R Square Change | F Change | df1 |
| 1 | ,180[a] | ,032 | -,056 | 1,184 | ,032 | ,367 | 5 |

**Model Summary**

| Model | Change Statistics df2 | Change Statistics Sig. F Change |
|---|---|---|
| 1 | 55 | ,869 |

a. Predictors: (Constant), number_of_CPR_courses, professional_experience, gender, profession, age

**ANOVA[a]**

| Model | | Sum of Squares | df | Mean Square | F | Sig. |
|---|---|---|---|---|---|---|
| 1 | Regression | 2,573 | 5 | ,515 | ,367 | ,869[b] |
| | Residual | 77,066 | 55 | 1,401 | | |
| | Total | 79,639 | 60 | | | |

a. Dependent Variable: correct_classification_CPR_only

b. Predictors: (Constant), number_of_CPR_courses, professional_experience, gender, profession, age

## Coefficients[a]

| Model | | Unstandardized Coefficients B | Std. Error | Standardized Coefficients Beta | t | Sig. |
|---|---|---|---|---|---|---|
| 1 | (Constant) | 6,172 | ,858 | | 7,190 | <,001 |
| | age | -,018 | ,032 | -,121 | -,557 | ,580 |
| | gender | -,448 | ,387 | -,169 | -1,156 | ,253 |
| | profession | ,097 | ,473 | ,042 | ,205 | ,838 |
| | professional_experience | -,016 | ,205 | -,015 | -,078 | ,938 |
| | number_of_CPR_courses | ,052 | ,131 | ,070 | ,400 | ,691 |

## Coefficients[a]

| Model | | 95,0% Confidence Interval for B Lower Bound | Upper Bound | Collinearity Statistics Tolerance | VIF |
|---|---|---|---|---|---|
| 1 | (Constant) | 4,452 | 7,892 | | |
| | age | -,083 | ,047 | ,374 | 2,675 |
| | gender | -1,224 | ,328 | ,826 | 1,211 |
| | profession | -,852 | 1,046 | ,410 | 2,438 |
| | professional_experience | -,426 | ,394 | ,481 | 2,079 |
| | number_of_CPR_courses | -,210 | ,315 | ,576 | 1,735 |

a. Dependent Variable: correct_classification_CPR_only

## Coefficient Correlations[a]

| Model | | | number_of_CPR_courses | professional_experience | gender |
|---|---|---|---|---|---|
| 1 | Correlations | number_of_CPR_courses | 1,000 | -,102 | -,060 |
| | | professional_experience | -,102 | 1,000 | ,213 |
| | | gender | -,060 | ,213 | 1,000 |
| | | profession | -,563 | ,218 | -,219 |
| | | age | ,085 | -,667 | ,039 |
| | Covariances | number_of_CPR_courses | ,017 | -,003 | -,003 |
| | | professional_experience | -,003 | ,042 | ,017 |
| | | gender | -,003 | ,017 | ,150 |
| | | profession | -,035 | ,021 | -,040 |
| | | age | ,000 | -,004 | ,000 |

## Coefficient Correlations[a]

| Model | | | profession | age |
|---|---|---|---|---|
| 1 | Correlations | number_of_CPR_courses | -,563 | ,085 |
| | | professional_experience | ,218 | -,667 |
| | | gender | -,219 | ,039 |
| | | profession | 1,000 | -,498 |
| | | age | -,498 | 1,000 |
| | Covariances | number_of_CPR_courses | -,035 | ,000 |
| | | professional_experience | ,021 | -,004 |
| | | gender | -,040 | ,000 |
| | | profession | ,224 | -,008 |
| | | age | -,008 | ,001 |

a. Dependent Variable: correct_classification_CPR_only

**Collinearity Diagnostics**[a]

| Model | Dimension | Eigenvalue | Condition Index | Variance Proportions (Constant) | age | gender | profession |
|---|---|---|---|---|---|---|---|
| 1 | 1 | 5,453 | 1,000 | ,00 | ,00 | ,00 | ,00 |
| | 2 | ,305 | 4,229 | ,01 | ,00 | ,01 | ,00 |
| | 3 | ,153 | 5,970 | ,00 | ,00 | ,31 | ,00 |
| | 4 | ,048 | 10,632 | ,00 | ,01 | ,31 | ,50 |
| | 5 | ,028 | 13,850 | ,67 | ,01 | ,34 | ,20 |
| | 6 | ,013 | 20,691 | ,32 | ,97 | ,04 | ,30 |

**Collinearity Diagnostics**[a]

| Model | Dimension | Variance Proportions professional_experience | number_of_CPR_courses |
|---|---|---|---|
| 1 | 1 | ,00 | ,01 |
| | 2 | ,02 | ,58 |
| | 3 | ,16 | ,02 |
| | 4 | ,20 | ,23 |
| | 5 | ,25 | ,15 |
| | 6 | ,37 | ,02 |

a. Dependent Variable: correct_classification_CPR_only
